# Share repurchase and the cost of capital: Discussion on the nature of share repurchase of Chinese listed companies

**DOI:** 10.1371/journal.pone.0292171

**Published:** 2023-09-28

**Authors:** Ping Wang, Rui Chen

**Affiliations:** Department of Accounting, Capital University of Economics and Business, Beijing, China; Shanghai Business School, CHINA

## Abstract

Share repurchase is not only an important financial policy of the company, but also a financial policy with a very wide range of influence. A scientific and reasonable share repurchase policy has a significant financial effect in terms of an equity structure optimization, capital structure adjustment, cash holding control, and even management compensation system design. The long-standing share repurchase in the U.K. and U.S. companies and the recent practice of share repurchase by Chinese listed companies imply an important financial proposition: share repurchase is a high-quality financial policy that, when properly applied, will help reduce the cost of capital. The data in this study show that share repurchases can reduce the level of firms’ cost of capital and play an important role in enhancing their competitiveness. This study also finds that Chinese listed companies, when formulating and implementing share repurchase programs, do not subjectively view share repurchases as a policy tool to adjust cash holdings and capital structure. In addition, they do not view share repurchases as an alternative to cash dividends but use equity incentives for management as the main intention for share repurchases. This decision-making concept is not conducive to the rational use of share repurchase policies in the long run and should be a cause for concern in the business community. Finally, the leverage and the cash holding path are the main motives affecting share repurchases and are the important fundamental mechanisms in this study, while the mediating mechanism test finds that share repurchases mainly reduce the cost of capital through two information perspectives of analyst following and stock liquidity. The heterogeneity analysis shows that the role of share repurchases in reducing the cost of capital is more obvious in enterprises with fierce product market competition, high institutional investor shareholding.

## 1. Introduction

Share repurchase is a financial policy with extremely complex connotations. Why should a company repurchase shares it issued? What is the relationship between share repurchases and cash dividends? How do share repurchases affect shareholder wealth? Similar to the well-known mysteries of capital structure and dividends, share repurchases have become an important financial puzzle [1].

The proliferation of share repurchases in countries such the U.K. and the U.S. began after the 1980s. In comparison, the large number of share repurchases by Chinese listed companies has been a recent event. According to the Wind Database (https://www.wind.com.cn/) of share repurchases of Chinese A-share listed companies, 1,925 companies announced 7,127 share repurchase programs from 2011 to 2021. The number of share repurchase programs surged from 633 to 1,338 especially from 2017 to 2018. Both from the perspective of corporate governance and management, share repurchases require scientific interpretation and analysis. Doing so will enhance the rationality and standardization of share repurchase policies and ensure the interests of investors as well as the long-term sustainable development of companies.

From the early literature, the academic community generally considers share repurchases by firms as a substitute for cash dividends [2–4]. However, an important feature of dividend policy is the need to maintain the stability of cash dividend payments. If a company holds a large amount of cash and no desirable investment opportunities exist, the company may distribute the excess cash holdings to shareholders through share repurchases without changing the dividend policy. This explanation is supported to some extent by data that show a declining trend in cash dividend payouts in conjunction with a gradual increase in share repurchases by companies [2]. Nonetheless, field research conducted on the business community finds significant differences from the academic view. Practitioners usually view share repurchases as an investment behavior [5, 6], that is, buying shares of a company at a lower price when the stock market is declining. This view is essentially consistent with how Chinese companies view share repurchase, which is a means of market capitalization management.

In studying share repurchases, two aspects must be distinguished: one is the source of funds for share repurchases, the other is the destination of share repurchases. From the perspective of the specific operation of domestic and foreign firms, two main sources of funds for share repurchases exist. One is to repurchase shares in cash, which will reduce the scale of assets, and the other is to repurchase shares in new liabilities, which will not affect the scale of assets but will affect the capital structure of the firm. Share repurchase, whether reducing cash holdings or changing capital structure, will affect the cost of capital and the wealth of investors. The destination of repurchased shares can be divided into destruction and reissue, such as using repurchased shares for equity incentives.

The nature of share repurchases is mainly determined by the source of funds rather than the destination. In other words, share repurchases should be studied in terms of their impact on cash holdings and capital structure. How the repurchased shares are disposed of is not a decisive factor affecting the nature of share repurchases and will not have a fundamental impact on the company and investors. This issue must be clarified in the study of share repurchases.

Unlike cash dividend policy, share repurchase is not an inclusive wealth distribution mechanism for corporate shareholders. Generally, shareholders who participate in a share repurchase program are always a small part of all shareholders, even very few equity holders. The most fundamental feature of the dividend policy is to treat all shareholders equally and achieve the goal of equal share and profit. If the share repurchase policy is examined only from the perspective of mutual substitution of share repurchase–cash dividend, it will obscure some basic features of this policy and not be conducive to the scientific formulation of the share repurchase policy. At the same time, this feature of share repurchases makes studying share repurchases directly from the perspective of investors or the market impossible.

The cost of capital is the rational rate of return required by investors based on the risk of investment. As a fundamental concept of corporate finance, the cost of capital has been studied for more than a century. During this long historical evolution, its important role in both the micro and macro fields has been gradually recognized. The symbolic and benchmarking role of cost of capital has gained increasing application in the field of micro studies. With other factors unchanged, a lower cost of capital implies higher quality of corporate governance, higher value creation, and stronger competitiveness. Conversely, a higher cost of capital implies poorer quality of corporate governance, lower value creation, and lower competitiveness.

That share repurchase will be a major financial policy that cannot be ignored during the development of a firm can be predicted. This study focuses on a fundamental question, is the share repurchase policy a good financial policy or a bad financial policy? According to the modern corporate finance theory, a good financial policy helps reduce the cost of capital, while a bad financial policy increases the cost of capital, and diminishes the wealth of shareholders. Research shows that share repurchase is a good financial policy from the perspective of cost of capital. As long as the share repurchase policy is reasonably formulated and implemented, the cost of capital can be reduced. This notion is consistent with the reality that share repurchases have been widely used in Chinese and foreign firms for decades.

The remainder of this paper is structured as follows: Section 2 reviews the relevant literature and develops the hypotheses. Section 3 depicts the descriptive statistics. Section 4 introduces the research design. Sections 5 reports the empirical results. Section 6 describe the robustness test. Sections 7 and 8 presents the impact mechanism test and additional tests, respectively. Section 9 conduct the heterogeneity test. Finally, Section 10 summarises the conclusions, contributions, and policy implications.

## 2. Related literature and hypothesis development

From the perspective of literature review, the study of share repurchase is related to that of dividend policy. In share repurchase research, the most critical issues are twofold, namely, (1) the motivation and (2) consequences of share repurchase. On the basis of these issues, the relationship between share repurchases and dividends is sought, that is, whether share repurchases are a substitute for dividends.

Share repurchases are not a new topic in corporate finance; they began in the U.S. corporate world in the 1980s. The widespread use of share repurchases is directly related to government regulation of capital markets and changes in the legal system. Intuitively, when the legal system and government regulations are not conducive to the distribution of cash dividends, companies seek alternative ways to pay out earnings to shareholders. For example, cash dividends may face higher tax burdens for investors. The numerous government constraints on cash dividend payouts that can limit a company’s dividend distribution. When the constraint of dividend payment conflicts with a company’s plan for shareholder returns, share repurchases may be applied as an alternative. This idea is one of the most straightforward in share repurchase research and has gained much recognition. In 1982, the U.S. Securities and Exchange Commission (SEC) implemented regulatory reform, which liberalized the anti-manipulation provisions of the Securities Exchange Act that had been in place since 1934 and adopted Rule 10B-18 [2]. In 1986, the U.S. introduced the Tax Reform Act, which abolished the special treatment of capital gains [4, 7]. In addition, the SEC issued a regulation 10B5-1 in 2000, which further evaded more insider trading charges about share repurchase for listed companies.

As share repurchases are increasingly widely used by the business community, it has become clear that the phenomenon is much more complex than expected in two ways. First, the nature of a dividend payment is a distribution of net income and can reduce the company’s cash holdings simultaneously. However, share repurchase is not necessarily a distribution of net income. In many cases, the company is buying back shares for the increased liability. At this time, the greatest impact of share repurchase is the capital structure rather than the change in the firm’s asset size, such as the reduction of cash holdings. Second, related to the first point, why does a firm repurchases shares? Is it a substitute for cash dividends? Or is it to improve the debt ratio and capital structure? Or is it just to get pure investment income? The implementation of the share repurchase program often has many consequences, which inevitably have a very significant compounding effect. This compounding effect leads to the diversification of research findings and increases the complexity of the share repurchase research.

The motives for the possible repurchases of shares discussed by the academic community are regulatory and tax considerations [4, 8], agency costs of free cash flow [6, 9], capital structure adjustment [10], acquisition defense [11], signal or undervaluation hypothesis [12, 13], substitute cash dividends [2], stock options [14], confiscation of wealth from bondholders [15] and excess cash distribution [9].

Are share repurchases a substitute for cash dividends? Based on different research perspectives, the answer to this question is also subject to significant disagreement. In *Disappearing dividends* [16], documented a sharp decline in the proportion of public companies paying dividends from 1978 to 1999. Deangelo et al. [3] showed that although the number of U.S. firms paying cash dividends decreased, the dollar value of the actual total dividends paid by the companies paying dividends actually increased. Grullon and Michaely [2], Hsieh and Wang [4], and Skinner [17] further pointed out that the substitution effect between cash dividends and share repurchases. In particular, some U.S. firms have subjectively replaced the distribution of cash dividends with share repurchases. Cash dividend payment is extremely rigid and may need to consider certain industry characteristics, which greatly restrict the flexibility of dividend policies. Share repurchases can essentially eliminate the constraints on the formulation of these policies and enhance management’s autonomy in providing compensation for shareholders. Some scholars also did not believe that these two payment methods can be substituted for each other [18, 19]. In addition, a large number of field studies show that the practitioners of corporate finance regard share repurchase more as an investment behavior [5, 6].

A concern in this study is the ultimate consequences of share repurchase behavior. From the perspectives of corporate governance and corporate finance, the most central issues are firm value and shareholder wealth, which are closely related to the cost of capital. According to the classic Modigliani-Miller theory, the cost of capital is the fundamental factor that determines the value of the firm and then the wealth of shareholders [20]. When other factors remain unchanged, the lower the cost of capital, the greater the value of the firm, and the more the wealth of shareholders. At the same time, the lower the cost of capital, the stronger the competitiveness. As an important financial policy, the implementation of a scientific and reasonable share repurchase programs must have a beneficial impact on the cost of capital. On the contrary, an unreasonable and failed share repurchase programs may be because the board of directors has given too much consideration to the interests of the controlling shareholders when formulating the plan, thus detracting from the wealth of the small and medium-sized shareholders. For example, in the Chinese stock market, some companies may in essence be transferring wealth to management when they implement share repurchases.

Theoretically and logically, share repurchases can reduce a company’s cost of capital through changes in (1) working capital policy and (2) capital structure policy. In particular, the focus of the share repurchase policy is not on the act of repurchase itself but on optimizing the working capital and capital structure policies through share repurchases. This is the main concern when formulating a share repurchase policy.

First, share repurchases optimize working capital policies and reduce the cost of capital by reducing cash holdings. They will directly reduce a company’s cash holdings. At the same time, the part of cash used for repurchases will constitute a return for shareholders. The historical evolution of this financial policy demonstrates that distributing excessive cash holdings to shareholders is an important reason for declaring share repurchases.

According to the free cash flow hypothesis, this behavior will help reduce agency conflicts and improve the quality of working capital management. On the one hand, free cash flow is distributed to shareholders to alleviate the conflict of interest between the management and shareholders [21–23] and prevent the use of capital ineffectiveness. Share repurchases are an important way to achieve this goal. On the other hand, the explanation for the free cash flow hypothesis supporting share repurchase is the positive reaction of the market to the repurchase announcement. Grullon and Michaely [2] provided evidence that announcing a share repurchase program leads to a very positive market reaction when a company’s investment opportunities decline. The three main points in time when a company announces a share repurchase program that is associated with share price volatility are as follows: (1) Repurchase activities are negatively correlated with the prior stock return, and companies repurchase shares when their stock price is undervalued by the market and there are excess cash distributions [12, 24]. (2) Companies that repurchase shares get significantly positive announcement returns [10]. (3) The overall effect between the company’s repurchase announcement and the share price may continue for several years [25, 26]. In other words, the share repurchase policy can be judged as a good financial policy in terms of signaling effects.

Second, share repurchases can reduce the cost of capital by optimizing a company’s capital structure. If the direct intention of share repurchases is to adjust the capital structure, share repurchases are often carried out by adding new liabilities, thus reducing the cost of equity capital while increasing liabilities and ultimately making a more substantial adjustment to the capital structure. This adjustment implies a higher debt ratio and a convergence to an optimal capital structure. Share repurchase has become a tool for dynamic adjustment of capital structure. On the one hand, share repurchase provides the management with a way to change the capital structure by buying back shares with additional liabilities while reducing the number of shares issued out of the company, reducing the cost of equity capital, and increasing leverage [27, 28]. Dittmar [10] also found that companies with low debt are more willing to increase the company leverage ratio by repurchasing shares. On the other hand, if the management sets an optimal target capital structure, management will hope to adjust the company’s leverage to the optimal value [16, 29, 30]. This is known as the dynamic adjustment hypothesis of financial leverage, which indicates that firms tend to adjust dynamically to achieve their target capital structure when they have additional debt accommodation capacity.

As previously stated, the nature of share repurchase is mainly determined by its source of funds. Different sources of funds determine whether the direct goal of share repurchase is to adjust the working capital policy or the capital structure policy, which means that the nature of share repurchase is determined. The purpose of share repurchases is only the subsequent operation of share repurchases, such as the destruction of shares, the use of shares for equity incentives, the reissue of shares. They are not relevant to the nature of the share repurchase.

In reality, most of the shares repurchased are used to implement a company’s equity incentive plan. Equity incentive plans can be very effective in dissolving agency conflicts between shareholders and management and are widely used by the business community [31–33]. Especially in China, the rational design and implementation of equity incentive plans have attracted more attention in recent years.

Based on the three motivations for share repurchases, we identify the fundamental mechanism through which share repurchases may reduce a firm’s cost of capital, and the complex mechanism of their effects is the focus of our discussion. Share repurchases have a significant announcement effect that is usually interpreted by the stock market as good news, and the announcement itself is sufficient to send a positive signal to current shareholders [12]. Through this signal, the investors interpret the repurchase as better news for some firms than others, thus creating a better information environment for the firm. In turn, share repurchase stabilizes the share price of the company and increases its stock liquidity [34]. In sum, companies declaring share repurchase programs can reduce their cost of capital through the two important fundamental mechanisms of lowering cash holdings and regulating capital structure. Share repurchases may reduce the level of a company’s cost of equity capital mainly by improving its information environment and increasing its information efficiency path. The basic hypothesis of this study is as follows:

***Hypothesis 1***: ***Share repurchases are negatively associated with the level of a company’s cost of capital***.

## 3. Descriptive statistics

### 3.1 Expected share repurchase program

The sample of share repurchases, the expected number of share repurchases, and the proportion of total share capital of listed Chinese A-share companies in each year from 2011 to 2021 are compiled based on the share repurchase data from the Wind Database to explore the situation of share repurchases since 2011. Given the small number of share repurchase programs announced in the early years, the sample of listed companies announcing share repurchase programs in China’s Shanghai and Shenzhen A-shares in the more ten-year period from 2011 to 2021 is used to mitigate the effect of other noise.

As shown in Table 1, in terms of time distribution, 1,925 companies announced 7,127 share repurchase programs from 2011 to 2021. Only four companies declared buybacks in 2021, whereas the sample increased from 31 buyback proposals to 109 buyback proposals from 2012 to 2013. Despite the increase in numbers, companies announced relatively few buyback proposals to the public during this period. From 2017 to 2018, there was a surge from 633 to 1,338 buyback proposals, twice as many as in 2017 and accounting for 19.641% of the total sample. A continued climb in the number of buybacks was witnessed in 2019, with 52 more compared to 2018. The following year (2020) saw a total of 1,243 repurchases, maintaining a high level. In 2021, the highest number of repurchases was reached with a total of 1,423. The expected number of share repurchases in the 7,127 share repurchases from 2011 to 2021 was 76,168 million shares. The expected number of repurchases increased to 15.005 billion shares in 2018 with the support of national policies, which is more than six times the shares in 2017. The repurchase scale was a record high. In the past three years, the number of repurchases was also in a high state, with the expected number of repurchases rising to 18.804 billion shares. However, the ratio of 0.945% of the total share capital is not as high as 1.040% in 2019. From 2020 to 2021, the figures were affected by external situations such as the COVID–19 outbreak, so the number of repurchases was slightly lower than in 2019, at 15.893 billion shares and 11.812 billion shares, respectively.

**Table 1:** Sample statistics on expected share repurchases for each year.

Year	share repurchase programs	Number of firms	Expected number of share repurchases	Proportion in total share capital
2011	4	4	0.473	1.763
2012	31	25	16.276	2.577
2013	109	69	6.978	0.785
2014	203	94	38.664	0.647
2015	305	126	33.521	0.687
2016	448	158	25.840	0.419
2017	633	165	24.788	0.408
2018	1338	466	150.049	1.040
2019	1390	344	188.039	0.945
2020	1243	214	158.928	0.699
2021	1423	260	118.119	0.673
Total/Avg.	7127	1925	122.685	0.770

Note: This table presents the sample statistics on expected share repeats for each year. Specifically, it shows the number of share repurchase programs, the number of firms, and the expected number of share repurchases, in 100 million shares, and the proportion of the total share capital.

The proportion of the expected number of share repurchases in the total share capital decreased from 1.763% in 2011 to 0.673% in 2021. The annual average share repurchases accounted for about 0.770% of the total share capital, reaching the sample maximum of 2.577% in 2012. This numbers illustrate that the share repurchase programs is not an inclusive behavior of firms to provide returns to shareholders, but the choice of some shareholders. This difference is the most important between the share repurchase policy and the cash dividend policy.

### 3.2 Successful share repurchase: The number and amount of repurchases

**Fig 1:**
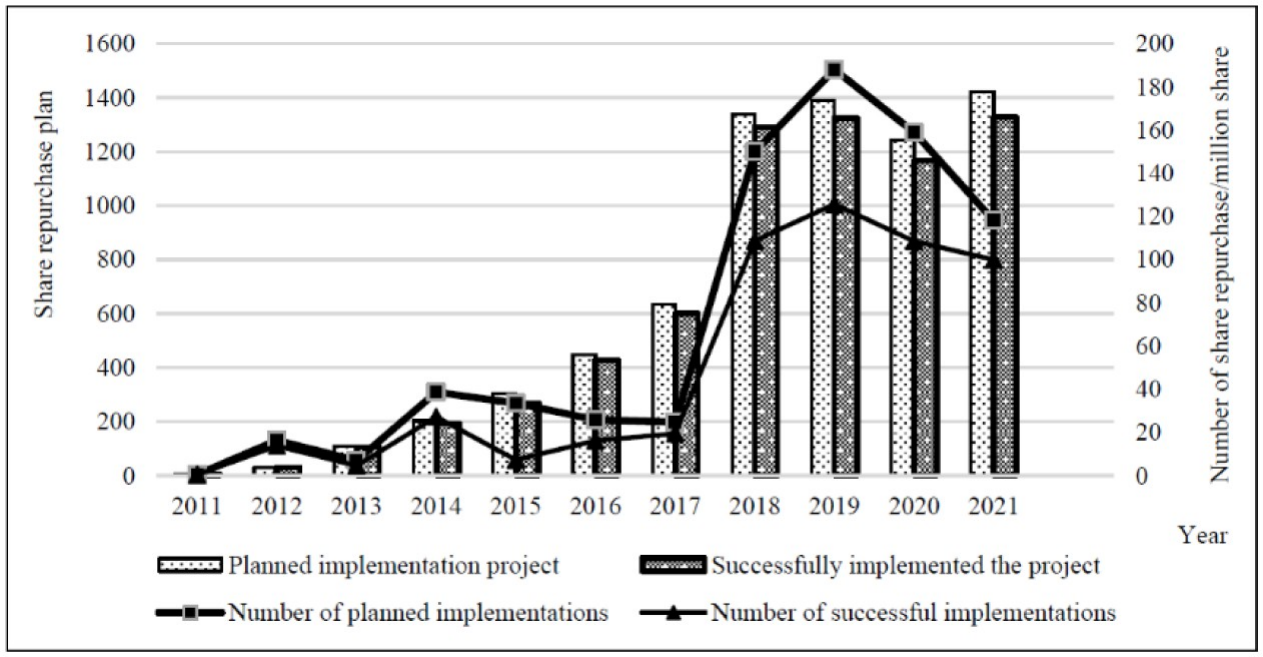
Comparison between share repurchase programs and successful implementation. Note: This table presents the comparison of share repurchase programs and successful implementation. Specifically, it shows planned implementation project, successfully implemented the project, number of planned implementations in 100 million shares and number of successful implementations in 100 million shares.

As shown in Table 2 and Fig 1, the total number of A-share listed companies that successfully completed share repurchases in 2011–2021 was 1,855, with 6,741 share repurchase programs completed, repurchasing 53.197 billion shares for 382.313 billion yuan. Seventy firms failed to complete the repurchase program and failed the program 386 times, with 22.971 billion more shares expected to be repurchased than successfully repurchased. The average ratio of shares successfully repurchased to total share capital was 0.533%, which was 0.237% lower than the ratio of shares expected to be repurchased to total share capital. Among them, 1,324 share repurchase programs were implemented by listed companies in 2019, with the number of shares repurchased being 12,570 million and the repurchase funds being 86,393 million yuan, which were historically high. In 2020, the number and amount of repurchases decreased compared with those in 2019. In 2021, 1,328 successful repurchases were made, the largest number of programs in the calendar year, but the number of repurchases of 9,997 million shares was not as high as the number of repurchases of 12,570 million shares in 2019. Yet, the repurchase amount was the highest of all years. These figures indicate that with the standardization and legalization of share repurchases, a growing number of companies focusing on completing the repurchases as pre-programmed without causing large-scale share repurchase default events.

**Table 2:** Sample statistics of successful share repurchases by Chinese listed companies in 2011–2021.

Year	Share repurchases programs	Number of firms	Expected number of share repurchases	Repurchase amount	Proportion in total share capital
2011	4	4	0.500	4.989	1.180
2012	30	24	13.770	67.483	0.766
2013	106	67	4.610	25.530	0.231
2014	193	88	27.196	78.587	0.176
2015	269	103	7.278	57.278	0.202
2016	428	150	16.164	70.005	0.198
2017	601	160	19.443	108.281	0.301
2018	1291	462	108.795	737.378	0.705
2019	1324	340	125.696	863.934	0.649
2020	1167	215	108.548	745.773	0.564
2021	1328	242	99.965	1,063.893	0.572
Total/Avg.	6741	1855	531.965	3823.131	0.533

Note: This table presents Sample statistics of successful share buybacks by A-share listed companies in China in 2011–2021. Specifically, it shows the number of share repurchase programs, the number of firms, and the expected number of share repurchases in 100 million shares, repurchase amount in 100 million yuan, and the proportion of the total share capital.

### 3.3 Sources of funding for share repurchases

Share repurchase funds come from cash held by the company or new debt. As previously discussed, the source of funding for share repurchases is the decisive factor in judging the nature of share repurchases. Reducing cash holdings while satisfying shareholder payout requirements is the main goal of share repurchase policy when repurchasing shares in cash. By contrast, increasing debt ratio and optimizing capital structure are the main goals of share repurchase policy when repurchasing shares with new debt.

Table 3 indicates that the main source of funding for share repurchases by Chinese A-share listed companies was still cash holdings from 2011 to 2021, with a total of 6,720 times, accounting for 94.462% of the total sample. New debt accounted for only 394 times. The percentage of repurchases using cash was extremely high, with repurchases totaling 40.441 billion shares and repurchases totaling 3110.068 billion shares from 2011 to 2021. The total number of repurchases using new debt amounted to 11.34 billion shares and the amount was 74.746 billion yuan, indicating that new debt occupies a certain scale in the share repurchase programs. In terms of trend, the number of repurchases completed with cash holdings was declining year by year, but the number of repurchases with new debt jumped from 0 shares in 2012 to 3.606 billion shares in 2020. Share repurchases using newly new debt can effectively improve corporate governance and enhance the operation efficiency of the overall capital market.

**Table 3:** Statistical source of funds for share repurchases of Chinese listed companies.

Year	Cash holding				New debt			
	Freq.	Percent	Number	Amount	Freq.	Percent	Number	Amount
2011	4	0.060	0.500	4.989	0	0.000	0.000	0.000
2012	29	0.432	13.538	65.885	2	0.508	0.232	1.598
2013	105	1.563	4.600	25.428	2	0.508	0.004	0.101
2014	196	2.917	2.987	15.423	2	0.508	0.383	5.679
2015	296	4.405	7.823	64.956	6	1.523	0.106	1.638
2016	441	6.563	17.022	80.450	5	1.269	0.064	1.730
2017	619	9.211	17.447	83.961	14	3.553	2.648	31.996
2018	1194	17.768	78.176	499.954	143	36.294	30.807	240.811
2019	1260	18.750	95.811	622.465	130	32.995	31.219	243.988
2020	1196	17.798	74.209	604.131	47	11.929	36.064	149.109
2021	1380	20.536	92.295	1,042.426	43	10.914	11.879	70.811
Total	6720	94.462	404.408	3110.068	394	5.538	113.406	747.461

Note: This table presents statistical source of funds for share repurchases of A-share listed companies in China. Of the 7127 repurchase information, 7114 disclosed the source of repurchase funds, and 13 were not disclosed, so they were eliminated when calculating the proportion. Specifically, it shows the two sources of share repurchase funds are cash held by the company or new debt. It presents the number of samples, percent, the number of share in 100 million shares and number of amount in 100 million yuan.

### 3.4 Purposes of share repurchases

In August 2019, Inner Mongolia Yili Industrial Group Co., Ltd. (600887.SH) repurchased shares at a high price and sold them to management at half price, giving an equity incentive of 183 million shares to 474 company employees and a lower threshold for equity incentive programs. Since 2006, Illy has declared four equity incentives programs, and multiple equity incentives have led to a gradual increase in Illy management’s shareholding ratio and increased control over the company. Share repurchase as an equity incentive for management has become one of the main features of the share repurchase program of Chinese listed companies.

From the perspective of the use of repurchase, the diversification trend of share repurchase purposes is obvious. The number of listed companies implementing repurchases for the purpose of implementing equity incentives, equity incentive cancellation, profit compensation, and market value management are increasing year by year. At present, the main purpose of share repurchase is to implement equity incentives and market value management.

**Fig 2:**
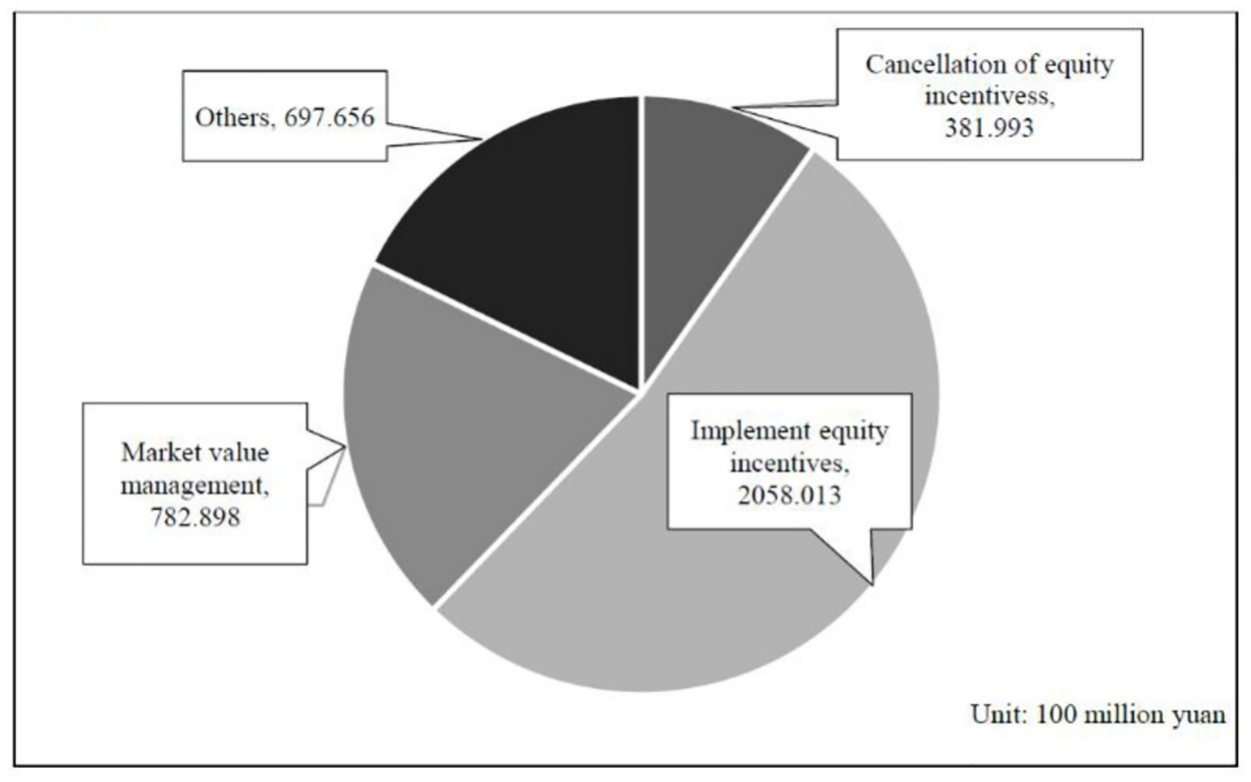
Statistical figure of share repurchases purposes of Chinese listed companies. Note: This figure shows a pie chart of the amount of share repurchases by listed companies in China for different purposes. In the different cuts, four categories are represented, namely, cancellation of equity incentives, implementation of equity incentives, market value management, and others. The unit is 100 million yuan. Data source: The data were obtained from the Wind database (https://www.wind.com.cn/) and the Resset database (http://www.resset.cn/), and the specific amount is shown in Table A2 (S1 Appendix), which is overall graphical and plotted by the authors to make it easier for readers to access useful data.

In listed companies in China, the incentive targets have changed. When the incentive targets face resignation, contract expiration, and non-renewal, layoff, and retirement, the restricted shares they have originally received but not yet released from the restriction on sale have to be written off by repurchase. As a result showed in Fig 2, the number of share repurchases for cancellation of equity incentives is relatively high from 2012 to 2021, which was 5,144 times, accounting for 72.176% of the total sample. However, the proportion of the number and amount of real repurchases is small and is not the main reason for repurchases by Chinese listed companies.

### 3.5 Percentage of net income after tax on cash payments: Cash dividends and share repurchases

Formally, cash dividends and share repurchases are transfers of firm value to shareholders through cash payments. In the absence of share repurchases, the proportion of cash payments in after-tax net profit is the so-called dividend payout ratio. If share repurchases are viewed as a substitute for cash dividends, as the amount of cash paid to shareholders through share repurchases increases, the amount of cash paid to shareholders through dividends tends to decrease. If the principle of the traditional dividend policy remains unchanged, the sum of cash paid by dividends and cash paid by share repurchases as a percentage of after-tax net profit remain at a relatively stable and reasonable level. The data for Chinese companies, do not support this claim. In other words, companies generally do not take the payment of cash dividends into account when implementing share repurchase.

**Fig 3:**
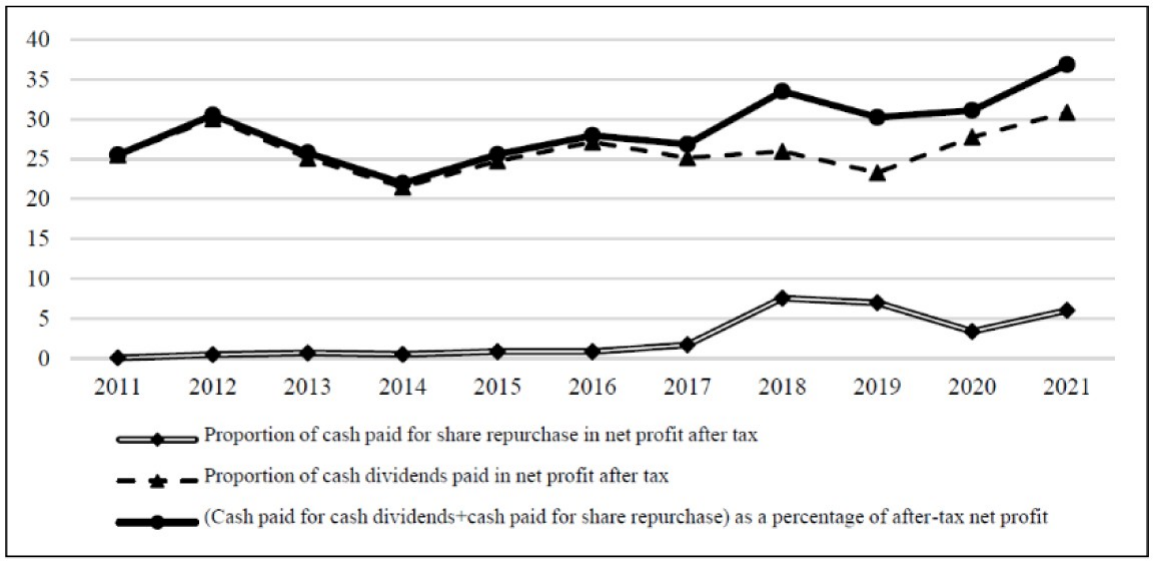
Statistics on the percentage of net profit after tax paid by Chinese listed companies in China, 2011–2021. Data source: share repurchases data is from Wind Database, cash dividend data is from CSMAR Database, and the graph is compiled by the author. The same below. Note: The samples used in this analysis only include companies with positive profits. In order to mitigate the impact of outliers, the observed values with payment ratio greater than 100% and less than 0 are eliminated here.

As shown in Table 4 and Fig 3, with reference to the practice of Grullon and Michaely [2], this study first calculates the proportion of cash paid by each company to after-tax net profit by share repurchases and cash dividends to investors. It then calculates the average cash payment rate of each year in the sample. The average cash paid for share repurchases accounted for 3.057% of the after-tax net profit in 2011–2011. From 2011 to 2017, the cash paid for share repurchases accounted for only a small proportion of the after-tax net profit. Then the share repurchases entered a stage of slow rise. In 2018, after China significantly lifted the restrictions on share repurchases, it ushered in a significant increase. The cash paid for share repurchases increased from 1.685% in 2017 to 7.536% of the net profit after taxes. In the past two years, the proportion began to decline slowly and remained at a certain high level. Unlike foreign companies, in the same period, the proportion of cash paid to shareholders through dividend policy in net profit did not change significantly with the increase in share repurchase. The proportion of cash dividends paid of after-tax net profit increased from 25.476% to 30.861% in 2011–2011, with an average payment ratio of 26.109%. The proportion of cash paid for cash dividends and cash paid for share repurchases in the after-tax net profit increased from 25.529% in 2011 to 36.863% in 2021, reflecting the increase in cash remuneration paid to shareholders during the decade. These data show that Chinese listed companies did not regard share repurchases as a substitute for cash dividends. Moreover, companies did not consider the change in shareholders’ wealth as an important factor when implementing share repurchase programs.

**Table 4:** Statistics on the percentage of net profits after tax on cash payment of Chinese listed companies in China, 2011–2021.

Year	Cash paid for share repurchase/net profit after tax (%)	Cash paid for Cash dividend /net profit after tax (%)	(Cash paid for share repurchase + Cash paid for Cash dividend)/net profit after tax (%)
2011	0.053	25.476	25.529
2012	0.450	30.066	30.516
2013	0.667	25.105	25.772
2014	0.494	21.470	21.964
2015	0.835	24.745	25.580
2016	0.827	27.127	27.954
2017	1.685	25.164	26.849
2018	7.536	25.961	33.497
2019	6.966	23.281	30.247
2020	3.351	27.748	31.099
2021	6.002	30.861	36.863
Avg.	3.057	26.109	29.166

Data source: Share repurchases data from the Wind Database, cash dividend data from the CSMAR Database, tabled by the author.

### 3.6 Impact of share repurchases on relevant financial indicators

In this analysis, four indicators are adopted to make a comparative argument for share repurchase. It is used to compare whether the annual trends of the repurchase amount of companies that successfully declared share repurchases are correlated with changes in cash holdings, capital structure adjustment, and market value change. It further explores the effect of share repurchase on Chinese listed companies, whether it is the adjustment of capital structure or the return to shareholders.

**Fig 4:**
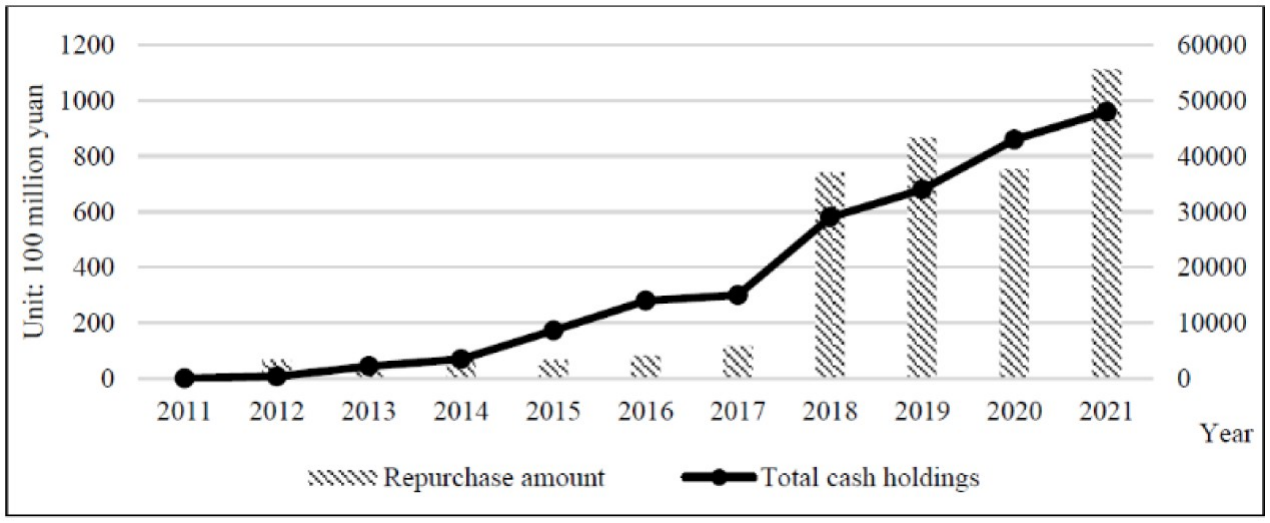
Comparison of repurchase amounts to total cash holdings.

The design of share repurchase programs is directly related to the extraordinary increase in cash holdings. Excessive cash holding not only means the waste of capital occupation but also causes serious agency problems. By implementing share repurchase, the company’s cash holdings can be moderately reduced and a reasonable level of working capital can be maintained. However, no direct and significant relationship between cash holdings and share repurchases is observed from the relevant data of Chinese listed companies. Therefore, cash holding is not an important consideration in the share repurchase policy. As shown in Table 5 and Fig 4, the repurchase amount and total cash holdings of repurchasing companies showed a year-on-year increase during 2011–2021, with a total repurchase amount of 392.056 billion yuan and total cash holdings of 1,977.326 billion yuan. The amount of share repurchase by companies was insignificant compared with their total cash holdings.

**Table 5:** Impact analysis of share repurchases in 2011–2021.

Year	Repurchase amount	Total cash holdings	Cash holding /Current assets	Asset-liability ratio	Market to book ratio
2011	4.989	36.159	35.032	42.455	8.507
2012	67.483	372.381	36.173	39.444	9.587
2013	25.530	2,210.782	32.487	34.432	14.676
2014	84.133	3,484.973	29.017	36.892	16.693
2015	66.593	8,628.264	30.985	37.077	23.677
2016	82.180	14,000.000	28.581	38.204	18.066
2017	115.957	15,000.000	28.044	39.010	16.361
2018	740.766	29,000.000	24.416	40.048	11.550
2019	866.453	34,000.000	24.448	41.522	19.440
2020	753.240	43,000.000	26.822	42.126	23.740
2021	1,113.236	48,000.000	26.873	42.535	30.073
Total/Avg.	3920.560	197732.600	26.506	40.684	20.456

Note: Repurchase amount unit: 100 million yuan; Total cash holdings = balance of cash and cash equivalents at the end of the period/10,000,000, unit: 100 million yuan; Cash holdings/current assets = balance of cash and cash equivalents at the end of the period/current assets * 100%; Asset-liability ratio = total liabilities/total assets * 100%, unit: %; Market to book ratio = market value/book value.

**Fig 5:**
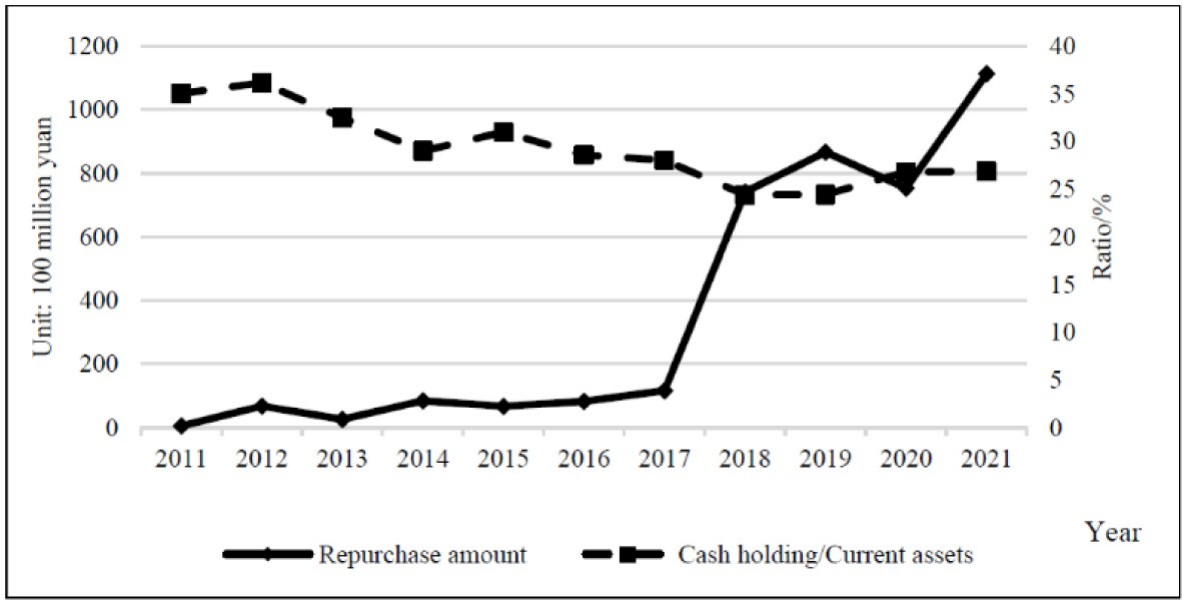
Comparison of repurchase amounts with cash holdings/current assets.

Fig 5 illustrates that cash holding/current assets decreased from 35.032% to 26.822% with an average ratio of 26.873% from 2011 to 2021, with a clear downward trend from 2012 to 2014. In 2018, it decreased to an annual low of 24.416% and then started to increase year by year. The use of cash to repurchase shares is decreasing year by year as share repurchases increase.

**Fig 6:**
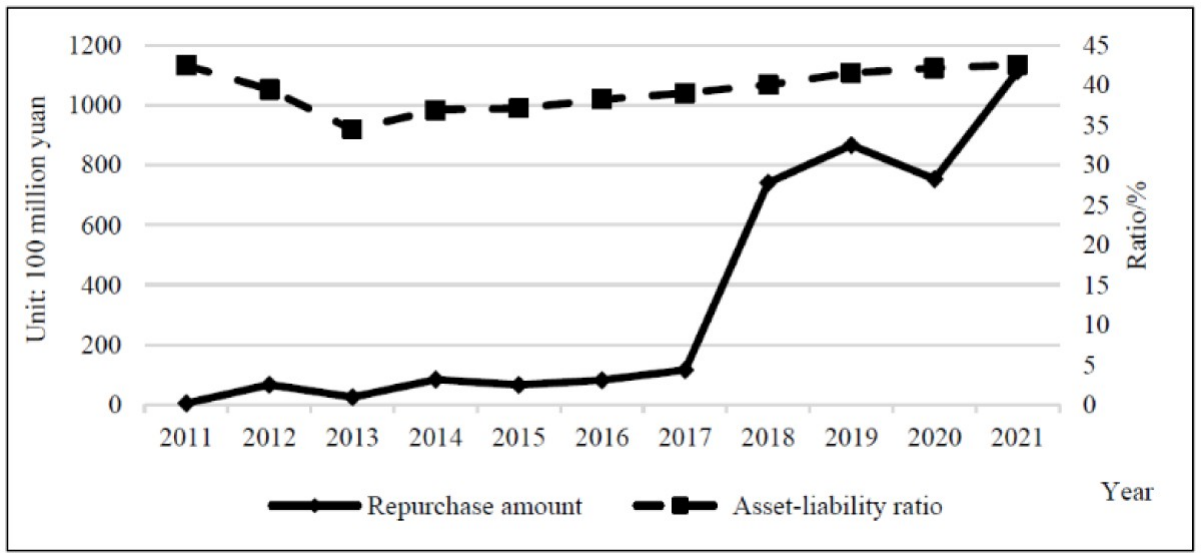
Comparison of repurchase amount to asset liability ratio.

Fig 6 depicts that the average asset–liability ratio changed from 42.455% in 2011 to 42.535% in 2021, and the average asset–liability ratio was 40.864%. From 2011 to 2013, an obvious downward trend was noted, falling to the annual minimum of 34.432%, but it began to rise year by year. The data shows that share repurchases have not become an important means for Chinese listed companies to adjust their capital structure policies.

**Fig 7:**
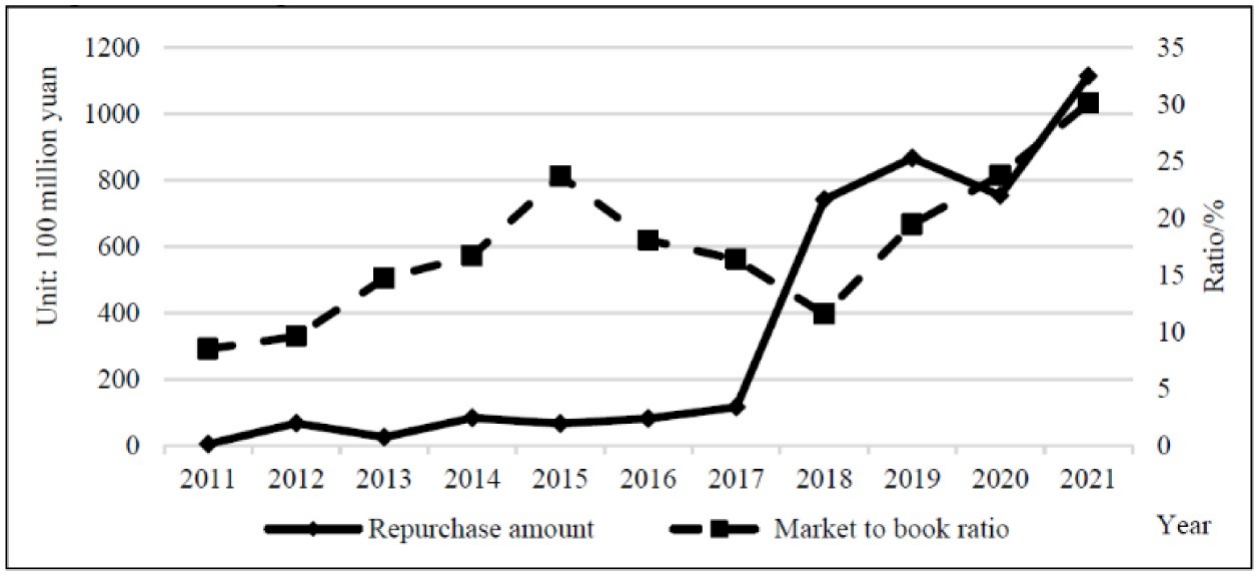
Comparison of repurchase amount to market to book ratio.

Under the same circumstances, an increase in the price-to-book ratio implies an improvement in the company’s market value and increase in shareholders’ wealth. A reasonable share repurchase should contribute to an increase in the price-to-book ratio on top of contributing to a lower cost of capital. The consequences of a share repurchase implemented by a company may also be possible to evaluate on the basis of the changes in the rise or fall of the price-to-book ratio. This is the basis on which Chinese companies try to achieve market value management purposes through share repurchases. Against the backdrop of sub-optimal capital market efficiency, achieving this goal can be fraught with variables and requires careful justification. The data support this point. Fig 7 shows that the average price-to-book ratio of repurchasing companies from 2011 to 2021 was 20.456, rising from 8.507 in 2011 to 30.073 in 2021. A significant increase was noted in the price-to-book ratio in 2015–2016 as the repurchase amount declined. However, the price-to-book ratio declined as the repurchase amount rose in 2018. The statistics confirms from one side that the repurchases of most companies in the year did not bring a good market reaction to the company.

### 3.7 Share repurchases as an alternative to cash dividends

A preliminary conclusion has been reached earlier that Chinese listed companies do not consider share buybacks as a substitute for cash dividends. A more detailed analysis is presented here.

The total sample is divided into four cases according to the companies’ payment policies. First, companies that pay cash dividends are set as DIV and companies that declare share repurchase programs are set as REPO. If a company pays cash dividends in 2011 and repurchases shares in the current year, it is classified as a company that pays cash dividends and share repurchases (i.e., DIV>0 and REPO>0), corresponding to the result in column (4) of Table 6. By analogy, all A-shares paying cash dividends and share repurchases from 2011 to 2021 are counted.

**Table 6:** Sample statistics on share repurchases and cash dividends by year, 2011–2021.

Year	(1)	(2)	(3)	(4)
	DIV = 0, REPO = 0	DIV>0, REPO = 0	DIV = 0, REPO>0	DIV>0, REPO>0
2011	867	1609	2	2
2012	807	1768	3	22
2013	1039	1789	11	68
2014	1342	1785	14	133
2015	1321	1785	29	184
2016	1203	2167	38	263
2017	1287	2451	53	358
2018	1565	1995	115	657
2019	1581	2140	167	605
2020	1201	2453	125	607
2021	817	2617	143	686
Total	13030	22559	700	3585

Column (1) of Table 6 shows that the sample of companies that neither pay cash dividends to shareholders nor declare share repurchases (DIV = 0, REPO = 0) fluctuated between 2011 and 2021. Hence, a number of companies that did not pay cash to shareholders. As the total number of companies increased from 807 in 2012 to 1,581 in 2019, the number of those companies that do not distribute has undergone different magnitudes of change and started to significantly decline since 2019. This phenomenon may also be explained by our Semi-Mandatory Dividend Policy and the enforcement of share repurchases. In column (2), the sample of companies that paid cash dividends to shareholders but did not make share repurchases (DIV>0, REPO = 0) includes 22,559 sample, occupying a larger proportion. A slow upward trend was observed from 2011 to 2016. The maximum value of the sample was reached in 2017 with 2,451 companies, followed by a slow downward trend. Thus, cash dividend payments remain the main way for Chinese companies to pay out earnings to shareholders. Column (3) focuses on the companies that did not pay cash dividends to shareholders but made share repurchases (DIV = 0, REPO>0). The sample had 700 companies in total, growing from 2 companies in 2011 to 143 companies in 2021. Column (4) shows the companies that paid cash dividends to shareholders and also made share repurchases (DIV>0, REPO>0). A total of 3,585 companies implemented such a payment policy during 11 years, growing from only 2 companies to 686, with the larger value of 657 companies in 2018. The statistics verifies that since the state encouraged share repurchases in 2018, the phenomenon has become more common in China and been gradually used by listed companies.

## 4. Research design

### 4.1 Data source and sample selection

Given that the sample of announced share repurchase programs is extremely small in the early years, the sample interval of this study is taken from the eleven years of listed Chinese companies in Shanghai and Shenzhen Stock Exchange in China from 2011 to 2021 in order to attenuate the influence of other noise, excluding newly listed companies in 2021, B-share companies, companies in the financial industry, companies with missing financial data, ST companies, and companies with negative cost of capital estimates. The valid cost of capital sample of 26,568 was obtained by screening. The share repurchase data are obtained from WIND database and RESSET database, the cost of capital data is obtained from the CSMAR and *Capital University of Economics and Business · Miller Salon’s Cost of Capital Estimation Database for Chinese Listed Companies in 2022*^1^. Additionally, by removing the top and bottom 1% of values for each variable^2^, the impact of outliers on the analysis is reduced, further improving the reliability of the results.

### 4.2 Variable setting

#### 4.1.1 Dependent variable

The use of *Specification Guidance on Cost of Capital Estimation for Listed Companies in China 2022*^3^ helps to ensure that the cost of equity capital estimates is reliable and accurate for Chinese listed companies. The use of implied cost of capital estimation metrics and HVZ data for each period, as recommended by Hou [35], further improves the accuracy of the estimates. The use of multiple models, including the Gordon model [36], CT model [37], OJ model [38], GLS model [39], PEG model and MPEG model [40], helps to ensure that the cost of equity capital estimates is robust and not overly influenced by any one model. Taking the arithmetic average of the estimates from these six models, further enhances the reliability and accuracy of the cost of equity capital estimates for the companies in the sample.

Overall, the use of a rigorous and comprehensive cost of equity capital estimation method helps to ensure that the results of the analysis are reliable and accurate, and can be used to inform important business decisions.

1. In the Gordon model, *P*_0_ uses the stock price in the previous period, *dsp*_1_ uses the dividend per share in the current period, and *g* represents the sustainable growth rate.
$$R_{e}=\frac{dps_{1}}{P_{0}}+g$$ (1)
2. In the CT model, *P*_0_ uses the prior-year closing price, *bps*0_*tz* is the adjusted net asset per share, *eps*_*t*_ is the first to five-year surplus per share from the cross-sectional regression forecast model, *bps*_*t*−1_ is the calculated “clean surplus” relationship, *bps*_*t*−1_ is the calculated prior-period dividend per share, and *g* is the rate of maturity for ten-year bonds.
$$p_{0=bps_{0}\_tz}+\sum_{t=1}^{s}\frac{eps_{t}-r_{e}\times bps_{t-1}}{{\left(1+r_{e}\right)}^{t}}+\frac{\frac{(eps_{5}-r_{e}\times bps_{4})\times \left(1+g\right)}{r_{e-}g}}{{\left(1+r_{e}\right)}^{5}}$$ (2)
3. In the OJ model, the predicted earnings per share in the first and second year, represented by *eps*_1_ and *eps*_2_, respectively, are estimated using a cross-sectional regression forecasting model. The long-term growth rate, represented by the parameter γ-1, is also an important factor in the model. In addition to these variables, the yield to maturity of the 10-year treasury bond is used as a factor. The closing price at the end of the previous year, is represented by *P*_0_, and *dps*_1_ represents analyst forecast data.
$$R_{e}=A+\sqrt{A^{2}+\frac{esp_{1}}{P_{0}}\times \left(\frac{esp_{2}-esp_{1}}{esp_{1}}-\left(\gamma -1\right)\right)}$$ (3)
  Among them, $\text{A}=\frac{1}{2}\left(\gamma -1+\frac{dps_{1}}{P_{0}}\right)$.
4. In the GLS model, *bps*_0_ it is the adjusted net assets per share, *P*_0_ represents the closing price at the end of the previous year, and *roe* represents the forecasted net income per share.
$$P_{0}=bps_{0}+\sum_{t=1}^{3}\frac{roe_{t}-R_{e}}{{\left(1+R_{e}\right)}^{t}}bps_{t-1}+\sum_{t=4}^{11}\frac{roe_{t}-R_{e}}{{\left(1+R_{e}\right)}^{t}}bps_{t-1}+\frac{roe_{12}-R_{e}}{R_{e}{\left(1+R_{e}\right)}^{11}}bps_{11}$$ (4)
5. In the PEG model, the predicted earnings per share in the first and second year, represented by *eps*_1_ and *eps*_2_, respectively, are estimated using a cross-sectional regression forecasting model. The closing price at the end of the previous year, represented by *P*_0_ is also an important factor.
$$R_{e}=\sqrt{\frac{esp_{2}-esp_{1}}{P_{0}}}$$ (5)
6. In the MPEG model, *dsp*_1_ is the predicted earnings per share. Other alphabetical variables are the same as the PEG model.
$$R_{e}=\sqrt{\frac{esp_{2}+R_{e}\cdot dsp_{1}-esp_{1}}{P_{0}}}$$ (6)

#### 4.2.2 Independent variable

Following Busch and Obernberger [41], Hillert et al. [42], Ota et al. [43], Hsu and Huang [44], and Lobo [45], we set up a dummy variable for share repurchases (*Rep_Dummy*) and a continuous variable (*Rep_Ratio*). *Rep_Dummy* represents whether the listed company repurchases or not. This variable equals 1 if the company announces a share repurchase plan and equals 0 otherwise. Meanwhile, we measure the planned size of the repurchase program (*Rep_Ratio*) by dividing the number of shares to be repurchased by the total number of shares outstanding.

#### 4.2.3 Control variables

We control for firm-level factors that prior studies identified as influencing a firm’s cost of equity capital (e.g., Dhaliwal et al. [46]; Fama and French [47]; Botosan and Plumlee [48]). We initially incorporate the Fama-French three factors [47], firm size (*Size*), M/B ratio (*Mtb*), and beta (*Beta*). Tseng and Demirkan [49] and Hong et al. [50] show that future growth opportunities (*Growth*) is negatively associated with cost of equity capital. Thus, we assume that a firm’s leverage (*Lev*) and operation risk level (*Oprisk*) are positively associated with the cost of equity capital [48, 50, 51]. Accordingly, we also control for these two factors. In addition, Hail and Leuz [52] find that the cost of equity capital is positively associated with stock return volatility (*Return*). Therefore, we control for stock return variability [53, 54]. We also control for cash holdings (*Cash*) to measure the abundance of cash [46]. Given that cash is an inverse measure of liquidity risk, we expect this coefficient estimate to be negative [55]. We also include year and industry fixed effects^5^ and cluster the standard errors by firm [56]. The control variables and their descriptions are shown in Table A1 (S1 Appendix).

#### 4.2.4 Empirical models

In order to analyze the one-way correlation between the firm’s share repurchase and the cost of capital, this study establishes models (7) and (8) to test the main assumptions:

$$Re_{i,t}=a_{0}+a_{1}Rep\text{\_}Dummy_{i,t}+a_{2}Controls_{i,t}+\sum Industry\;+\sum Year+\varepsilon_{i,t}$$ (7)

$$Re_{i,t}=a_{0}+a_{1}Rep\text{\_}Ratio_{i,t}+a_{2}Controls_{i,t}+\sum Industry\;+\sum Year+\varepsilon_{i,t}$$ (8)

In the model (7), $\;R_{e}{}_{{}_{i,t}}$ denotes the cost of equity capital of listed companies; *Rep_Dummy*_*i*,*t*_ denotes whether listed companies announce share repurchase programs, and if the regression coefficient *a*_1_ is significantly negative, which means that companies implementing share repurchase can reduce the cost of equity capital; *Controls*_*i*,*t*_ indicates control variable; *Industry* and *Year* are fixed effects of industry and year respectively; *ε*_*i*,*t*_ denotes the residual item, and the standard errors are adjusted for firm-level cluster clustering considering the panel data characteristics. Also in model 8, *Rep*_*Ratio*_*i*,*t*_ represents the proportion of share repurchases, and if regression coefficient *a*_1_ is significantly negative, which means that the higher the proportion of share repurchases, the lower the firm’s cost of equity capital.

## 5. Empirical results and analysis

### 5.1 Descriptive statistics of main variables

This study makes descriptive statistics on the relevant variables of share repurchases and the cost of equity capital of Chinese listed companies during the sample period. Table 7 shows the results of *R*_*e*_ show that the average value is 0.090, which is consistent with the findings of Lamoreaux et al. [57] and Richardson et al. [58]. The average value of *Rep_Dummy* is 0.155, and the standard deviation is 0.362, which indicates that 15.5% of listed companies announced share repurchases and that the sample has a large difference. The share repurchase ratio (*Rep_Ratio*) has an average of 0.127. Among the control variables, *Size*, *Bm*, and other variables are in line with normal distribution.

**Table 7:** Descriptive statistics of main variables.

Variables	Obs	Mean	Median	Sd.	Min	Max
*R*_*e*_	26568	0.090	0.085	0.033	0.032	0.185
*Rep_Dummy*	26568	0.155	0.000	0.362	0.000	1.000
*Rep_Ratio*	26568	0.127	0.000	0.530	0.000	3.680
*Size*	26568	22.251	22.063	1.306	19.936	26.298
*Bm*	26568	3.427	2.588	2.791	0.545	16.618
*Beta*	26568	1.045	1.055	0.295	0.309	1.886
*Growth*	26568	3.741	3.617	1.014	1.673	6.880
*Lev*	26568	0.411	0.403	0.199	0.053	0.857
*Oprisk*	26568	0.023	0.013	0.032	0.001	0.209
*Return*	26568	0.124	0.000	0.493	-0.554	2.130
*Cash*	26568	0.308	0.195	0.334	0.021	1.986

### 5.2 Empirical result

Table 8 reports the results of the multiple regressions of listed companies’ share repurchase behavior on the cost of capital, with columns (1)–(2) using the share repurchase dummy variable (*Rep_Dummy*) and the proportion of share repurchases (*Rep_Ratio*) as independent variables, respectively. Results show that the regression coefficients of whether the listed companies conduct share repurchases (*Rep_Dummy*) and the proportion of share repurchases (*Rep_Ratio*) on the cost of capital (*R*_*e*_) are –0.006 and –0.002, respectively, both of which are significant at the 1% level. In this case, conducting share repurchases can effectively reduce the level of cost of capital, hence supporting the hypothesis that share repurchases are negatively associated with a company’s cost of capital. This finding is also consistent with the results of previous studies [2, 59–61]. In terms of economic significance, the change in share repurchases from 0 to 1 reduces the average cost of capital by 1.401% [(−0.006×0.362)/0.155]. The coefficient estimates in Model (8) imply that on average, a one standard deviation increases in the share repurchase ratio (*Rep_Ratio*) leads to a 0.835% [(-0.002 × 0.530)/0.127] decrease in the cost of capital relative to the sample mean. These results indicate that share repurchase can significantly reduce the cost of capital of companies in both statistical and economic senses, thus verifying our main hypothesis.

**Table 8:** Regression analysis of share repurchase and the cost of capital.

	(1)	(2)
*Rep_Dummy*	-0.006***	
	(-11.257)	
*Rep_Ratio*		-0.002***
		(-6.245)
*Size*	-0.017***	-0.017***
	(-38.897)	(-39.059)
*Mtb*	-0.003***	-0.003***
	(-20.535)	(-20.820)
*Beta*	-0.004***	-0.004***
	(-5.194)	(-5.142)
*Growth*	-0.008***	-0.008***
	(-29.528)	(-29.156)
*Lev*	0.063***	0.063***
	(35.473)	(35.597)
*Oprisk*	0.104***	0.103***
	(15.240)	(15.201)
*Return*	0.018***	0.017***
	(36.999)	(36.655)
*Cash*	0.006***	0.006***
	(8.026)	(8.200)
*_cons*	0.460***	0.461***
	(47.565)	(47.725)
*Industry*	Yes	Yes
*Year*	Yes	Yes
*N*	26568	26568
*adj*. *R*^*2*^	0.450	0.447
*F-value*	364.88***	358.78***

Note: All t values in this table are adjusted by the standard error of cluster at the company and annual level.

*, * * and * * * represent the significance level of 10%, 5% and 1% respectively (the same as the following table).

As for our control variables, consistent with previous research, we find that firms with higher operational risk, leverage, and stock return variability face higher equity financing costs. This finding is supported by extant theories and previous studies [48, 53]. Moreover, a higher information availability, which is proxied by firm size, decreases the equity risk premium. This result is consistent with those of Fama and French [47] and Gebhardt et al. [39]. We also find a negative and statistically significant coefficient on *Mtb*, and *Growth*, thus confirming that high growth prospects help to estimate equity risk premium.

## 6. Robustness tests

### 6.1 Generalized matrix estimation (GMM)

Share repurchases and cost of capital may interact with each other to have an endogeneity problem of reverse causality. First, the DWH test is used to verify the existence of the endogeneity problem. Then, drawing on Hillert et al. [42], and Nguyen et al. [62], the price ceiling (*Repuplus*) and the average price of repurchase (*Repumean*) as instrumental variables, thereby using GMM to replace the OLS estimation of the main hypothesis model. Table 9 presents the GMM regression results. The coefficients of both instrumental variables in column (1) are positively correlated with *Rep_Dummy*, which indicates a strong correlation between the instrumental variables and the dependent variables. The coefficient of *Rep_Ratio* in column (2) is also positively correlated and significant at the 1% level. In addition, *Hansen J* in the overidentification test is greater than 0.05, thus also fulfilling the exogeneity hypothesis. Finally, after using instrumental variables as well as GMM estimation, our hypotheses are further supported.

**Table 9:** Endogenous test and GMM estimation of share repurchase and the cost of capital.

	*R*_*e*_	*R*_*e*_
*Rep_Dummy*	-0.007***	
	(-12.128)	
*Rep_Ratio*		-0.008***
		(-11.476)
*_cons*	0.459***	0.461***
	(47.547)	(47.966)
*Controls*	Yes	Yes
*Industry*	Yes	Yes
*Year*	Yes	Yes
*N*	26568	26568
*adj*. *R2*	0.450	0.438
*F-value*	366.25***	362.98***
*DWH test*	7.009***	15.294***
*Kleibergen-Paap rk Wald F*	1.2e+04	723.509
*Shea’s Adj*. *Partial R-sq*	0.782	0.276
*Minimum eigenvalue statistic*	47776.6	5073.21
*10% maximal IV size*	19.93	19.93
*Hansen J*	0.291	0.877

### 6.2 Heckman two-stage model test

To overcome the deviation caused by the sample self-selection of companies that declare share repurchases and companies that do not declare share repurchases, Heckman [63] two-stage regression model is adopted, and the following model is set up.

Phase I:

$$\begin{array}{l} \text{Pr}(Rep\_Dummy_{i,t}=1)=a_{0}+a_{1}MeanRep_{i,t-1}\text{+}a_{2}Dividend_{i,t-1}\text{+}a_{3}Agency_{i,t-1}\\ \text{+}a_{4}Share_{i,t-1}\text{+}a_{5}Tax_{i,t-1}\text{+}a_{6}Controls_{i,t-1}+\sum Industry+\sum Year\text{+}\varepsilon_{i,t} \end{array}$$ (9)

Phase II:

$$Re_{i,t}=a_{0}+a_{1}Rep\text{\_}Dummy_{i,t}\text{+}a_{2}IMR\text{+}a_{3}Controls_{i,t}+\sum Industry\;+\sum Year+\varepsilon_{i,t}$$ (10)

Phase II:

$$Re_{i,t}=a_{0}+a_{1}Rep\_Ratio_{i,t}\text{+}a_{2}IMR\text{+}a_{3}Controls_{i,t}+\sum Industry\;+\sum Year+\varepsilon_{i,t}$$ (11)

Model (9) is the first−stage, and the dependent variable *Rep*_*Dummy*_*i*,*t*_ is whether the firms declare share repurchases. Referring to Caton et al. [64], including not only added the control variables of the model (7) and (8), but also other factors that may influence the share repurchase decision, including cash dividend payment (*Dividend*_*i*,*t*−1_), management expense rate (*Agency*_*i*,*t*−1_), equity incentive intensity (*Share*_*i*,*t*−1_), and tax (*Tax*_*i*,*t*−1_), all of which are lagged. In particular, referring to Lennox et al. [65] to add the exclusively constraint (*MeanRep*_*i*,*t*−1_), which is defined as the proportion of share repurchases by other firms in the same industry and the same year. Model (9)−(11) is the second−stage model, which mainly tests the impact of share repurchase on the cost of capital. The inverse mills ratio (*IMR*) obtained from the Probit regression of model (9) is added to model (7) and (8) for regression analysis, and model (10) and (11) is obtained. The regression results of the Heckman two-stage model are shown in Table 10.

**Table 10:** Heckman two-stage test results.

	(1)	(2)	(3)
*MeanRep*_*t*−1_	1.860***		
	(27.939)		
*Divdend*_*t*−1_	0.137***		
	(3.327)		
*Agency*_*t*−1_	2.900***		
	(6.252)		
*Share*_*t*−1_	3.842***		
	(46.802)		
*Taxes*_*t*−1_	-0.776***		
	(-5.840)		
*Rep*_*Dummy*_*t*_		-0.003***	
		(-4.887)	
*Rep*_*Ratio*_*t*_			-0.001***
			(-3.520)
*IMR*		0.005***	0.007***
		(8.248)	(9.930)
*_cons*	-2.190***	0.393***	0.390***
	(-7.390)	(33.752)	(33.603)
*Control*_*t*−1_	Yes	No	No
*Control*_*t*_	No	Yes	Yes
*Industry*	No	Yes	Yes
*Year*	No	Yes	Yes
*N*	22447	22447	22447
*Rseudo R*^*2*^*/adj*. *R*^*2*^	0.192	0.486	0.486
*LR chi2/ F-value*	3906.11***	327.38***	327.12***

Column (1) in Table 10 is the regression result of the first stage, which shows that the exclusivity constraint (*MeanRep*_*i*,*t*−1_) is significantly positively correlated at the level of 1%. Therefore, the proportion of share repurchases of other companies in the same industry and the same year will affect the decision of share repurchases, which is consistent with the conditions for the selection of the variable. From the results of Heckman’s second stage of regression in column (2)–(3), *IMR* is significantly positive at the level of 1%. The model has endogenous problems caused by sample selection bias. After adding *IMR* as the control variable, the coefficient of *Rep_Dummy*_*i*,*t*_ and *Rep_Ratio*_*i*,*t*_ remains significantly negative, indicating that the conclusion that share repurchase can effectively reduce the cost of capital is still valid after controlling for the endogeneity problem caused by the sample self-selection bias. The study findings are now more reliable^6^.

### 6.3 Propensity score matching (PSM)

The potential for selective bias issues when comparing firms that declare share repurchases and firms that do not declare share repurchases. Therefore, we use a PSM method to control possible endogenous problems. The companies that declare share repurchases (*Rep* = 1) are used as the processing group, and a series of control variables (*Size*, *Mtb*, *Lev*, *Growth*, *Industry*, *Year*) included in the principal regression are used as the covariates of the first-stage logit regression. The 1:4 nearest neighbor matching and 0.01 caliper matching were used to find the control group with similar characteristics for the treatment group, and the matched samples were used for regression analysis^7^. Column (1)–(2) in Table 11 is the regression result of the sample after PSM, in which the average processing effect (ATT) of capital cost is –0.0062 and –0.0015, respectively, which is significant at the level of 1%. This shows that the level of capital cost that have announced share repurchase programs is 0.006 lower than that of other listed companies with similar characteristics. The regression results remain robust even after controlling for the selection bias of sample features, which further supports the conclusions drawn in the study. In other words, the paper suggests that the findings are reliable and not affected by sample selection bias.

**Table 11:** PSM estimation results.

	(1)	(2)
*Rep_Dummy*	-0.006***	
	(-10.102)	
*Rep_Ratio*		-0.002***
		(-4.956)
*_cons*	0.542***	0.545***
	(44.216)	(44.482)
*Controls*	Yes	Yes
*Industry*	Yes	Yes
*Year*	Yes	Yes
*N*	13881	13881
*adj*. *R*^*2*^	0.516	0.512
*F-value*	233.91***	227.26***
ATT	Difference = -0.006***; T = -9.040	

### 6.4 Fixed effect

We try to control the basic factors that affect the dependent variable, we introduce fixed effects of industry, and year in the model to control features that do not change with *Industry*, and *Year* systems. We also continue to cluster and adjust standard errors for firm. However, there may still be endogenous issues caused by missing variables. Therefore, the fixed effect of Firm and Year is further controlled in the model to exclude the effect of firm and annual events. As shown in Table 12 column (1)–(2), the regression coefficients of *Rep_Dummy* and *Rep_Ratio* are significantly positively correlated, which can prove the conclusion of this study.

**Table 12:** Fixed effect test results.

	(1)	(2)
*Rep_Dummy*	-0.005***	
	(-8.981)	
*Rep_Ratio*		-0.001***
		(-2.658)
*_cons*	0.541***	0.549***
	(36.829)	(37.349)
*Controls*	Yes	Yes
*Firm*	Yes	Yes
*Year*	Yes	Yes
*N*	13881	13881
*Within*. *R*^*2*^	0.412	0.409
*F-value*	233.91***	658.54***

### 6.5 Alternative measures of the cost of capital

In the previous test, the calculation of cost of capital is measured by HVZ. In this part of the test, the predicted value of Modified RI is used for measurement^7^ [66, 67], that is, the OJ models is selected to re-estimate the cost of equity capital (*R*_*e*_1), which is substituted into the model (7) and (8). It can be seen in column (1)–(2) of Table 13 that the conclusion of the model with original variables is consistent after inspection.

**Table 13:** Robustness test results.

	(1)	(2)	(3)	(4)
*Rep_Dummy*	-0.004***			
	(-7.015)			
*Rep_Ratio*		-0.001**		
		(-2.383)		
*Rep_Ratio1*			-0.003***	
			(-6.119)	
*Rep_Amount*				-0.000***
				(-11.174)
*_cons*	0.486***	0.487***	0.461***	0.459***
	(49.207)	(49.291)	(47.684)	(47.462)
*Controls*	Yes	Yes	Yes	Yes
*Industry*	Yes	Yes	Yes	Yes
*Year*	Yes	Yes	Yes	Yes
*N*	24198	24198	26568	26568
*adj*. *R*^*2*^	0.516	0.515	0.447	0.450
*F-value*	572.91***	564.77***	357.91***	364.24***

### 6.6 Alternative measures of share repurchases

By referring to the measurement of a share repurchase by Hillert et al. [42], we can quantify the share repurchase index, that is, generate *Rep_Ratio1* and *Rep_Amount*, and substitute the variable into the model (7), which *Rep_Ratio1* is defined as the proportion of the share repurchase to total share capital implemented at the end of the year. *Rep_Amount* is the natural logarithm of share repurchase amount. The regression results are shown in column (3)–(4) of Table 13, which still proves that share repurchases can reduce the cost of capital.

### 6.7 Change sample period

After the revision of the share repurchase system in the *Company Law* in 2018, the company has more autonomy in using the share repurchase as a financial instrument, and can also maximize its effectiveness, making the number of companies issuing the repurchase program surge and the proposed repurchase scale larger. In order to enhance the robustness of the conclusion, this paper changed the sample interval between 2011–2017 and 2018–2021 and re-regressed the model (7) and (8). The regression results are shown in Table 14. The coefficient of *Rep_Dummy* and *Rep_Ratio* is still significantly negative, indicating that the above conclusions can still be supported after changing the sample interval.

**Table 14:** Change sample period results.

	(1)	(2)	(3)	(4)
*Rep_Dummy*	-0.007***	-0.005***		
	(-9.908)	(-8.069)		
*Rep_Ratio*			-0.003***	-0.002***
			(-4.258)	(-5.437)
*_cons*	0.284***	0.647***	0.284***	0.648***
	(25.920)	(58.174)	(25.864)	(58.243)
*Controls*	Yes	Yes	Yes	Yes
*Industry*	Yes	Yes	Yes	Yes
*Year*	Yes	Yes	Yes	Yes
*N*	15041	11516	15041	11516
*adj*. *R*^*2*^	0.302	0.527	0.299	0.525
*F-value*	179.22***	230.66***	176.00***	228.78***

## 7. Impact mechanism test

Based on the analysis in the hypothesis section, we propose that the mechanism through which share repurchases reduce the cost of capital is based on their motivation, that is, share repurchases can reduce cash holdings [9] and adjust the capital structure [10]. This fundamental mechanism is important for firms. To explore the mechanisms through which two companies’ share repurchase intentions affect the cost of capital, we explain the two impact mechanisms from the perspectives of information [68], information environment [69], and information efficiency [42].

### 7.1 Leverage path of corporate fundamentals mechanism

The fundamental determinants of stock value are firm fundamentals, of which corporate leverage is an important factor in determining stock value. To test the influence of leverage path on share repurchase motivation, we divide our sample into high and low groups according to the mean annual leverage ratio and conducts regressions using model (7) and (8), respectively. Columns (1)–(4) of Table 15 show that share repurchases can effectively reduce the level of cost of capital, and a lower leverage ratio corresponds to a more significant effect of share repurchases on the reduction of the cost of capital. Companies with low leverage can effectively use the financial leverage effect of repurchases to improve their operating efficiency and performance, which in turn help to reduce the cost of capital. However, companies with higher leverage finance their share repurchases using free funds, which can lead to a free cash flow shortage and reduce the company’s solvency, thus indirectly weakening the effect of share repurchases on reducing the cost of capital. The results of the Chow test show that the between-group difference in the coefficient of share repurchase is significant at the 1% level.

**Table 15:** Estimated results of corporate fundamentals mechanism.

	Leverage path				Cash holding path			
	(1)	(2)	(3)	(4)	(5)	(6)	(7)	(8)
	*Lev* = 1	*Lev* = 0	*Lev* = 1	*Lev* = 0	*Cash* = 1	*Cash* = 0	*Cash* = 1	*Cash* = 0
*Rep_Dummy*	-0.005***	-0.006***			-0.007***	-0.006***		
	(-6.355)	(-8.429)			(-7.585)	(-8.846)		
*Rep_Ratio*			-0.002***	-0.002***			-0.002***	-0.002***
			(-3.566)	(-4.397)			(-3.696)	(-4.946)
*_cons*	0.397***	0.579***	0.399***	0.580***	0.509***	0.445***	0.512***	0.446***
	(35.787)	(34.506)	(35.990)	(34.485)	(35.104)	(41.314)	(35.276)	(41.431)
*Controls*	Yes	Yes	Yes	Yes	Yes	Yes	Yes	Yes
*Industry*	Yes	Yes	Yes	Yes	Yes	Yes	Yes	Yes
*Year*	Yes	Yes	Yes	Yes	Yes	Yes	Yes	Yes
*N*	13106	13462	13106	13462	8703	17865	8703	17865
*adj*. *R*^*2*^	0.429	0.531	0.427	0.529	0.477	0.444	0.474	0.441
*F-value*	173.16***	236.97***	171.80***	231.54***	129.66***	244.66***	129.99***	239.57***
*Chow test*	9.68***		7.15***		5.51***		3.50**	

### 7.2 Cash holding path of corporate fundamentals mechanism

The path of cash holdings may likewise influence the reducing effect of share repurchases on the cost of capital. Columns (5)–(8) of Table 15 show that the greater the firm’s cash holdings, the more significant the effect of share repurchases on the reduction of the cost of capital. Having excessive cash holdings is one of the direct motivations for companies to make share repurchases. This motivation also alleviates agency conflicts between management and shareholders, prevents the inefficient use of capital, supports the free cash flow hypothesis of share repurchases, and brings about a positive market response to repurchase announcements, which is highly conducive to reducing the company’s cost of capital. The result also passes the chow test.

Share repurchase may reduce the companies’ cost of capital by attracting analyst attention and improving the environment of enterprises [69] or effective of their information [42]. Therefore, we use analyst following and stock liquidity as proxies of our intermediary variables.

### 7.3 Information environment path

Following Sun and Sun [69], we use the number of analysts following to measure a firm’s information environment. We measure analysts following by the natural logarithm of the number of analysts followed plus 1. The missing values for the number of analysts following are replaced by 0. Following Baron and Kenny [70], we construct and test the following intermediary effect model:

$$Re_{i,t}=a_{0}+a_{1}Rep_{i,t}\text{+}a_{j}Controls_{i,t}+\sum Industry\;+\sum Year+\varepsilon_{it}$$ (12)

$$Analyst_{i,t}=b_{0}+b_{1}Rep_{i,t}\text{+}b_{j}Controls_{i,t}+\sum Industry\;+\sum Year+\varepsilon_{it}$$ (13)

$$Re_{i,t}=c_{0}+c_{1}Rep_{i,t}\text{+}c_{2}Analyst_{i,t}\text{+}c_{j}Controls_{i,t}+\sum Industry\;+\sum Year+\varepsilon_{it}$$ (14)

Where *Rep*_*i*,*t*_ is either *Rep*_*Dummy*_*i*,*t*_ or *Rep*_*Ratio*_*i*,*t*_. We test models (12)–(14) in turn. If the intermediary variable (*Analyst*) plays an intermediary role in the relationship between share repurchase and cost of capital, then coefficient *a*_1_ in model (12), coefficient *b*_1_ in model (13), and coefficient *c*_2_ in model (14) are all significant. Furthermore, when *c*_1_ is not significant, a complete intermediary effect occurs. When *c*_2_ is significant and when *b*_1_*c*_2_ and *c*_1_ have the same sign, a partial intermediary effect occurs. However, when these variables have different signs, a masking effect occurs.

The regression results of the intermediary effect of the number of analysts following (*Analyst*) are shown in Table 16. The coefficient *b*_1_ of the independent variable to the intermediary variable in column (2) is significantly positive, which suggests that the company’s share repurchase will increase the number of analysts following. Meanwhile, the coefficients of the independent and intermediary variables in column (3) to the independent variable *c*_1_ and *c*_2_ are also significant. The same sign of coefficients *b*_1_*c*_2_ and *c*_1_ indicates that increasing the number of analysts following has a partial intermediary effect on the relationship between share repurchase and cost of capital. Through the information environment Bootstrap test^8^, the direct effect interval (-0.005, -0.004), and (-0.002, -0.0001), which does not contain 0, further confirms the existence of a partial effect. Therefore, the implementation of share repurchase programs will reduce the cost of equity capital of the company by improving its information environment.

**Table 16:** Estimated results of information environment impact mechanism.

**Panel A**			
	**(1)**	**(2)**	**(3)**
	***R***_***e***_	***Analyst***	***R***_***e***_
*Rep_Dummy*	-0.006***	0.396***	-0.004***
	(-11.257)	(18.211)	(-8.359)
*Analyst*			-0.004***
			(-18.862)
*_cons*	0.460***	-9.607***	0.418***
	(47.565)	(-33.834)	(41.693)
*Controls*	Yes	Yes	Yes
*Industry*	Yes	Yes	Yes
*Year*	Yes	Yes	Yes
*N*	26568	26568	26568
*adj*. *R*^*2*^	0.450	0.430	0.463
*F-value*	364.88***	268.48***	382.68***
Bootstrap	95% Conf. Interval: (-0.005, -0.004)		
**Panel B**			
	**(1)**	**(2)**	**(3)**
	***R***_***e***_	***Analyst***	***R***_***e***_
*Rep_Ratio*	-0.002***	0.060***	-0.002***
	(-6.245)	(4.688)	(-5.454)
*Analyst*			-0.005***
			(-19.876)
*_cons*	0.461***	-9.717***	0.417***
	(47.725)	(-33.665)	(41.657)
*Controls*	Yes	Yes	Yes
*Industry*	Yes	Yes	Yes
*Year*	Yes	Yes	Yes
*N*	26568	26568	26568
*adj*. *R*^*2*^	0.447	0.417	0.462
*F-value*	358.78***	242.71***	381.81***
Bootstrap	95% Conf. Interval: (-0.002, -0.0001)		

### 7.4 Information efficiency path

Following Amihud [71], we use the non–liquidity index (*Amihud*_*i*,*t*_) as our proxy index of stock liquidity. The formula is shown in model (15):

$$Amihud_{i,t}=\frac{1}{D}\overset{D_{it}}{\underset{d=1}{\sum }}\left(\frac{\left|r_{itd}\right|}{V_{itd}}\right)\times 10^{6}$$ (15)

Where *r*_*itd*_ in model (14) represents the individual stock return of firm *i* on day *d* of year *t*, and *V*_*itd*_ represents the trading amount of firm *i* on day *d* of year *t*. *D*_*it*_ represents the transaction days of firm *i* on day *d* of year *t*, and |*r*_*itd*_| represents the change of stock return per unit of trading volume. The non-liquidity index is obtained by multiplying the average trading days by 10^6^. This indicator is a reverse indicator, where a higher value corresponds to a worse stock liquidity. We also add *Amihud*_*it*_ as an intermediary variable to models (12)–(14) for the regression test. The setting of these models are the same as those indicated in the above the information environment path.

Table 17 presents the regression results of the stock liquidity intermediary effect. The coefficient *b*_1_ of the independent variable to the intermediary variable in column (2) is significant and negative, indicating that share repurchase is positively related to stock liquidity. The coefficient of the independent and intermediary variables in column (3) to independent variable *c*_1_ and *c*_2_ are also significant, where *b*_1_*c*_2_, and *c*_1_ have the same sign. Therefore, increasing corporate stock liquidity has a partial intermediary effect on the relationship between share repurchase and the cost of capital. Through the information environment Bootstrap test, the direct effect interval (-0.006, -0.004), and (-0.002, -0.001), which does not contain 0, further confirms the existence of a partial effect. In sum, the implementation of share repurchase programs reduces the cost of equity capital of the company by increasing stock liquidity.

**Table 17:** Estimated results of information efficiency impact mechanism.

**Panel A**			
	**(1)**	**(2)**	**(3)**
	***R***_***e***_	***Amihud***	***R***_***e***_
*Rep_Dummy*	-0.006***	-0.001***	-0.005***
	(-11.257)	(-16.890)	(-9.482)
*Amihud*			1.638***
			(22.871)
*_cons*	0.460***	0.010***	0.443***
	(47.565)	(17.231)	(46.788)
*Controls*	Yes	Yes	Yes
*Industry*	Yes	Yes	Yes
*Year*	Yes	Yes	Yes
*N*	26568	26568	26568
*adj*. *R*^*2*^	0.450	0.111	0.477
*F-value*	364.88***	40.63***	414.98***
Bootstrap	95% Conf. Interval: (-0.006, -0.004)		
**Panel B**			
	**(1)**	**(2)**	**(3)**
	***R***_***e***_	***Amihud***	***R***_***e***_
*Rep_Ratio*	-0.002***	-0.000***	-0.002***
	(-6.245)	(-4.937)	(-5.944)
*Amihud*			1.667***
			(23.280)
*_cons*	0.461***	0.010***	0.445***
	(47.725)	(17.467)	(46.913)
*Controls*	Yes	Yes	Yes
*Industry*	Yes	Yes	Yes
*Year*	Yes	Yes	Yes
*N*	26568	26568	26568
*adj*. *R*^*2*^	0.447	0.108	0.475
*F-value*	358.78***	40.06***	412.69***
Bootstrap	95% Conf. Interval: (-0.002, -0.001)		

## 8. Additional tests

### 8.1 Share repurchases as an alternative to cash dividends

As mentioned earlier, cash dividend has been regarded as a substitute for share repurchase in Western countries [16]. However, in the previous descriptive statistical analysis, this relationship does not exist in China and that these are two separate policies. This study further uses regression analysis to explore the effect of cash dividends on the relationship between share repurchase and the cost of capital. Referring to Nguyen et al. [62], who used the cash dividend payout rate to measure the cash dividend (*Div*), we set the interaction term (Rep_Dummy*Div, Rep_Ratio*Div) of cash dividend and share repurchase. The regression results are shown in column (1)–(2) of Table 18. The coefficient of the interaction term is not significant, indicating that the impact of cash dividend on share repurchase and the cost of capital is not significant.

**Table 18:** The additional test of cash dividends regression results.

	(1)	(2)	(3)	(4)
Rep_Dummy*Div	-0.000			
	(-0.123)			
Rep_Ratio*Div		-0.000		
		(-0.073)		
*Rep_Dummy*	-0.006***		-0.006***	
	(-8.273)		(-11.176)	
*Rep_Ratio*		-0.002***		-0.002***
		(-4.208)		(-6.163)
*Div*	-0.002**	-0.002**	-0.002**	-0.002***
	(-2.237)	(-2.535)	(-2.430)	(-2.613)
*_cons*	0.459***	0.461***	0.459***	0.461***
	(47.429)	(47.585)	(47.435)	(47.587)
*Controls*	Yes	Yes	Yes	Yes
*Industry*	Yes	Yes	Yes	Yes
*Year*	Yes	Yes	Yes	Yes
*N*	26568	26568	26568	26568
*adj*. *R*^*2*^	0.450	0.447	0.450	0.447
*F-value*	348.22***	341.58***	355.71***	349.68***

In column (3)–(4) of Table 18, we re–estimate model (7) and (8), taking cash dividend (*Div*) as an additional control variable. We examine whether the impact of share repurchase on the cost of capital is affected by cash dividend. The result shows that the coefficient of share repurchase is still significantly negative, and the result does not change. Therefore, after controlling the cash dividend variable, the impact of cash dividend on share repurchase and the cost of capital can be excluded.

### 8.2 Different characteristics of share repurchase

We further investigate the effect of different characteristics of share repurchases on the cost of equity capital in terms of repurchase funding source, repurchase purpose, and repurchase mechanism.

#### 8.2.1 Repurchase funding source

The sources of funding for share repurchases are divided into own funds, bank loans, asset swaps, and equity for debt. Similarly, the effect of different sources of funds on the cost of equity capital varies. We consolidate the sources of funds for share repurchases into two categories, namely, self-financing repurchases and self-funded repurchases. Self-financing repurchase is the case of listed companies through borrowing, while self-funded repurchase refers to the use of a company’s own funds to repurchase shares. To observe the impact of share repurchase behavior on the cost of equity capital under these two sources, we set the source of repurchase funds (*Rep_Fund*) as our dependent variable, which equals 1 when firms use self-financing repurchases and 0 when they use self-funded repurchase. Given the small size of each subgroup after dividing share repurchases into different sources of funds, we select all control variables in model (7) as matching variables and perform PSM 1:4, caliper 0.01 sample matching according to the principle of nearest neighbor matching ^9^. We continue the regression using the samples are matched. The results in column (1) of Table 19 show that the coefficient of using self-financing repurchase (*Rep_Fund*) is negative and significant at the 1% level, indicating that the use of self-financing repurchase by firms reduces the cost of equity capital.

**Table 19:** Different characteristics of share repurchases and the cost of capital.

	(1)	(2)	(3)
*Rep_ Fund*	-0.008***		
	(-2.912)		
*Rep_Purpose*		-0.004***	
		(-3.947)	
*Rep_Mechanism*			-0.004***
			(-4.520)
*_cons*	0.471***	0.412***	0.467***
	(10.449)	(23.230)	(30.314)
*Controls*	Yes	Yes	Yes
*Industry*	Yes	Yes	Yes
*Year*	Yes	Yes	Yes
*N*	443	3269	5504
*adj*. *R*^*2*^	0.536	0.421	0.448
*F-value*	21.24***	55.07***	86.26***

#### 8.2.2 Repurchase purpose

According to the data form the Wind database are presented in the previous section, share repurchases have different purposes, including earnings compensation, equity incentives, market value management, and implementation or cancellation related to equity incentives. According to Gu and Xin [72], share repurchases in China are classified into active and passive repurchases. Active repurchases serve equity incentive, market value management, and earnings compensation motives, while passive repurchases serve all other motives, including equity incentive cancellation and restructuring^10^. The purpose of share repurchases reflects the willingness of a firm to repurchase to a certain extent with active repurchase indicating a slightly stronger willingness compared with passive repurchase. Active repurchase is motivated by equity incentive, while passive repurchase is motivated by equity incentive cancellation. Equity incentive is predicted to have an important role in reducing the cost of equity capital. Moreover, equity incentive programs in active repurchases give buyback commitments not only to outside investors but also to the incentive recipients, and market capitalization management repurchases are able to boost the company’s share price against external market-induced pressures [73]. To observe the impact of share repurchase behavior on the cost of equity capital under these two share repurchase purposes, we set repurchase purposes (*Rep_Purpose*) as our dependent variable, which equals 1 and 0 when the firm uses active and passive repurchases, respectively. Using PSM 1:4, caliper 0.01 sample matching, we continue our regression using the matched sample on model (7). The results in column (2) of Table 19 show that the coefficient of active repurchase (*Rep_Purpose*) is negative and significant at the 1% level, indicating that implementing active repurchases reduces the cost of equity capital.

#### 8.2.3 Repurchase mechanism

The share repurchase mechanism broadly includes both open-market and targeted repurchases. In practice, a listed company conducting share repurchases may use only open-market repurchases or only targeted repurchases in a year or may use both mechanisms. This section looks at the two mechanisms of share repurchases separately and their different impacts on the cost of equity capital. When a listed company conducts share repurchases in the current year through both open-market and targeted repurchases, we classify this situation as open-market repurchase because it is more likely to provoke a market response than a targeted repurchases and is closely related to investors’ interests. To observe the impact of share repurchase behavior on the cost of equity capital under these two share repurchase mechanisms, we set the repurchase mechanism (*Rep_Mechanism*) as our independent variable, which equals 1 and 0 when the firm repurchases through the open-market and targeted repurchases mechanism, respectively. After using PSM 1:4, caliper 0.01 sample matching, we continue the regression using the matched sample for model (7). The results in column (3) of Table 19 show that the coefficient of the open-market repurchases mechanism (*Rep_Mechanism*) is negative and significant at the 1% level, indicating that this mechanism reduces the cost of equity capital.

## 9. Heterogeneity test

### 9.1 Product market competition

We regard product market competition as the external governance mechanism of firms. With the intensification of product market competition, enterprises’ capital demand, financing demands and cash flow sensitivity increase; their internal financing capacity decreases; and the marginal value of companies’ cash holdings grows [74]. In addition, market competition can play an information role. Through information disclosure and other mechanisms, it can effectively improve the transparency of enterprises, thus reducing the alienation of insider behavior. Managers’ behavior of using capital holdings to seek private interests and other inefficient investments is inhibited, which is consistent with the goal of maximizing shareholder value and reducing the cost of capital of enterprises. Finally, product market competition is regarded as the most powerful driving force for enterprises to improve economic efficiency, which will promote enterprises to continuously improve and evolve under external environmental pressure, help effectively solve potential information and incentive problems, and improve corporate governance issues to a certain extent [75]. Therefore, under the condition of higher competition, the positive value effect of cash holdings is enhanced. The higher the quality of information disclosure is, the better the corporate governance is, and the better the external governance effect is achieved. Hence, the negative relationship between share repurchases and the cost of capital becomes more significant.

This study tests the product market competition with reference to Gioia [76] by setting the Herfindal–Hechiman index (*HHI*). The square sum of the operating revenue of each company in the industry as a proportion of the total operating revenue of the industry is used to measure the product market competition. The higher the value of the index, the lower the degree of competition. On the contrary, the higher the degree of competition is. This study takes the reciprocal of the index, divides it by 100, and replaces the interaction term (Rep_Dummy*HHI, Rep_Ratio*HHI) into the model (7) and (8). The regression results are listed in column (1)–(2) of Table 20. The regression coefficient of the interaction term to the cost of capital is –0.0002 and –0.0001, respectively, which is statistically significant. The reduction effect of share repurchase on the cost of capital is more obvious in regions where the product market competition is encouraged.

**Table 20:** Regression results of heterogeneity test.

	(1)	(2)	(3)	(4)
Rep_Dummy*HHI	-0.000*			
	(-1.908)			
Rep_Ratio*HHI		-0.000*		
		(-1.841)		
*Rep_Dummy*	-0.004***		-0.004***	
	(-4.193)		(-5.168)	
*Rep_Ratio*		-0.001*		-0.001***
		(-1.850)		(-2.747)
*HHI*	0.000***			
	(2.804)			
Rep_Dummy*Ins			-0.000***	
			(-3.631)	
Rep_Ratio*Ins				-0.000*
				(-1.761)
*Ins*			-0.001***	-0.001***
			(-12.773)	(-14.430)
*_cons*	0.000***	0.000**	0.438***	0.440***
	(2.804)	(2.537)	(45.136)	(45.385)
*Controls*	Yes	Yes	Yes	Yes
*Industry*	Yes	Yes	Yes	Yes
*Year*	Yes	Yes	Yes	Yes
*N*	26568	26568	26568	26568
*adj*. *R*^*2*^	0.450	0.447	0.460	0.458
*F-value*	356.73***	350.06***	376.39***	372.54***

### 9.2 Institutional investors

Institutional investors help investors judge the long-term valuation of the company based on their diversified investment decision–making ability and information collection and analysis advantages. To a certain extent, they play the role of supervision and management, alleviate the agency conflict between firms and investors, and improve information transparency [77, 78]. Changes in the concentration of institutional investors’ shareholding ratio will lead to subsequent changes in incentive compensation, thus acting as a regulator (Hartzell and Starks, 2003) [79]. The higher the shareholding ratio of institutional investors, the smaller the deviation between the company’s target capital structure and the actual capital structure, and the faster the company adjusts its capital structure. Therefore, the supervision mechanism of institutional investors can better play the role of corporate governance, alleviate the conflict between shareholders and managers, and optimize the company’s dynamic capital structure decision–making [80]. The supervision and governance of institutional investors will affect the company’s equity incentive plan and optimize the company’s capital structure decision, so the company’s share repurchase will also have a more obvious effect on reducing the cost of capital.

We use the institutional investor shareholding ratio (*Ins*) variable to verify this logic and generate the interaction term (Rep_Dummy*Ins, Rep_Ratio*Ins) of share repurchase and institutional investor shareholding ratio into the model (7) and (8). Column (3)–(4) of Table 20 show that the regression coefficient of the interaction term is –0.0031 and –0.0012, respectively, which is statistically significant. Therefore, the reduction effect of share repurchase on the cost of capital is significant in enterprises with high proportion of institutional investors.

## 10. Conclusions, contributions, and policy implications

The share repurchase policy is a high-quality financial policy with important applications in capital structure adjustment and cash holding control. From the perspective of optimizing corporate governance and management, the board of directors and management should have an accurate and profound understanding of share repurchase and implement this policy at the right time. Using data from Chinese listed companies, this study verifies the positive effect of share repurchases on the cost of capital. It also identifies several issues that require special attention: (1) Share repurchases are not a substitute for cash dividends in Chinese listed companies. In cases where management does not regard share repurchases as a reasonable alternative to cash dividends, the main application scenario of share repurchases is changed to create an equity base for management’s equity incentives. This is the main reason share repurchase programs have been widely developed and implemented by Chinese listed companies in recent years. In a practical sense, nothing is wrong with using the repurchased shares for management’s equity incentives. However, if the disposal of this repurchased shares is seen as an important or even the only value of the share repurchase policy, it will inevitably lead to the misuse and abuse of the share repurchase policy, which will then become an important tool for controlling shareholders to erode small and medium shareholders. This is a tendency that must be highly concerned. (2) Share repurchases have a significant financial effect. Through share repurchases, capital structure and cash holdings can be efficiently adjusted, thus reducing the cost of capital. A good share repurchase policy must achieve beneficial goals in terms of the financial effects of share repurchases, which is a basic principle of financial policy formulation and implementation. The formulation of any financial policy must be based on long-term objectives and should contribute to a sustainable increase in corporate value. Chinese listed companies still need to make more efforts in this regard.

Our study offers several contributions. First, we provide a better understanding of the motivations and determinants of share repurchases, which have not attracted much attention in the literature. Share repurchases initially emerged in Europe and the U.S. due to regulatory and tax considerations [4, 8], agency costs of free cash flow [6, 9], and substitute cash dividends [2]. However, previous studies fail to explain the true motivation of share repurchases and clarify their turning point. To fill this gap, we explore the true motivation of share repurchases in China, an emerging economy, following the 2018 amendments to the Company Law. We also provide a realistic contextual explanation for the turning point of share repurchase motives and establish a fundamental mechanism for studying the impact of share repurchases on the cost of capital in terms of cash holding motives for share repurchases and recapitalization motives, thus effectively revealing the nature of share repurchases of Chinese listed companies. This study differs from previous studies that focus on market capitalization management and undervaluation yet ignore the real purpose of the share repurchases of Chinese listed companies.

Second, previous studies on the microeconomic consequences of share repurchases merely focused on the production and innovation activities [62], liquidity, and accounting conservatism of firms [45], and only a few scholars have adopted the corporate finance perspective to explore debt financing or bond ratings [60, 61, 81, 82]. As the first empirical study to consider the cost of capital as an economic consequence, this study also provides positive empirical evidence from the capital market for ongoing share repurchases in China and a reference for studying share repurchase behavior in developing countries, thus complementing the literature and offering a new research perspective on the economic consequences of corporate share repurchases and cost of capital. We also elucidate the impact of share repurchases on cost of capital from two information perspectives, namely, information environment and information efficiency, based on the fundamental mechanisms of firms. In this way, our study extends the information chain of share repurchases and complements the findings related to the mechanism of improving the information environment and information efficiency in which firms operate [42, 68, 69].

Third, given that stock repurchases have become very common in emerging markets in recent years [83, 84], unlike previous studies that focus on the U.S. and the U.K., we locate our study in a country with significantly different cultural and institutional characteristics. Specifically, we provide direct evidence on the relationship between stock repurchases and financing in China. Our findings on Chinese listed companies are similar to those on firms in the U.K. and U.S. [44, 85, 86]. Our results may also be extended to developing countries and emerging markets and highlight time-varying effects of regulatory changes in emerging markets. These results may even help regulators further improve the laws and regulations related to share repurchases, differentiate the regulation of share repurchases for various funding sources, purposes, and mechanisms, and provide insights into the chain of interests behind a series of corporate behaviors. Authorities can then make changes to policies that can guide the repurchase behavior of listed companies and strictly control market risk. This study also provides a realistic reference for investors to understand the essence of share repurchases and recognize the investment risks and expected rewards and helps management to improving their corporate governance, which is of great practical significance for their realization of shareholders’ wealth.

This study provides a theoretical basis for regulators to formulate relevant policies, helps listed companies solve the financial mystery of share repurchase, and provides a reference for investors to understand corporate financial decisions in depth.

For regulators, on the one hand, the policy should guide and encourage companies with abundant cash flow and large deviations in the capital structure to conduct share repurchases so as to contribute to the healthy development of the capital market. Moreover, when the market is in the doldrums, the cancellation of repurchased shares by such companies will further highlight their core investment value and competitiveness, which is conducive to the injection of more long-term strategic capital, thus boosting investor confidence. On the other hand, regulators should improve their risk warning and precision management capabilities to accurately identify market risk states. To this end, they should strengthen the authenticity review of share repurchases by listed companies, strictly enforce the relevant regulations on information disclosure, and improve the quality of information disclosure requirements on the actual financial status and financing capacity of listed companies before and during share repurchases. In addition, they should urge listed companies to follow their ability to declare the amount and intensity of share repurchases and prevent companies from making excessive use of share repurchases to hide false information. Regulators should focus on listed companies that announce share repurchases repeatedly and continuously and analyze their market value, repurchase motives, and financial capacity to protect the interests of small and medium shareholders and ensure the stable and orderly development of the capital market.

That share repurchases are a good financial tool for firms has been documented. First, when listed companies have more idle capital and lack good investment opportunities, they can reduce the free cash flow of enterprises through share repurchases and alleviate their agency costs. Second, when enterprises complete share repurchases by raising debt, they can adjust their capital structure, thus reducing the gap between the actual capital structure and the target capital structure of the company; Thirdly, regarding their financing behavior, enterprises can set appropriate long-term incentive mechanisms, for example, increasing management shareholding or implementing stock option plans. By implementing long–term incentives, managers’ interests and shareholders’ interests can be closely linked to prevent their opportunistic behaviors and alleviate the serious principal–agent problem of the company, which affects the efficiency of corporate financing. To this end, companies can consider establishing a treasury stock system to provide them with the shares needed to implement their equity incentive plans. In this way, the expansion of share repurchases by listed companies becomes relevant to their financing policies.

For individual investors, their limited understanding of capital market phenomena and corporate financial behavior, as well as the late implementation of China’s share repurchase policy, leads to a lack of in-depth understanding of the repurchase mechanism. This study fully explores the nature of share repurchases of listed companies, which can enhance investors’ knowledge of the real motives of share repurchases of listed companies. The findings can prompt investors to allocate resources rationally in the capital market and improve investment efficiency and investment returns. Investors should also improve their information screening ability and pay attention to the financial data and share repurchase motives of companies implementing share repurchase programs so as to gain a more comprehensive and in-depth understanding of company management. Ultimately, doing all these help investors price the generated returns correctly, avoid risks, and protect their own interests.

## 11. Notes

1. The cost of capital data is obtained from the Capital University of Economics and Business· Miller Salon Cost of Capital Estimation Database for Chinese Listed Companies in 2022, which was estimated on November 16, 2022 by the Miller Salon team consisting of two teachers, Prof. Wang, Prof. Zou, and dozens of graduate students, combining the reality of the data provided by CSMAR database, Wind database, and RESSET database for Chinese listed companies (Wang, 2018) [87].
2. In order to ensure that the descriptive statistics and analytical results of share repurchase in the section 3 are accuracy and stable, the following major variables and regression results are not winsored in this study.
3. Specification Guidance on Cost of Capital Estimation for Listed Companies in China 2022 is from the Capital University of Economics and Business· Miller Salon. For a detailed description of the estimated the cost of capital of Chinese public companies (Wang, 2018) [87].
4. The industry classification results of listed companies for the third quarter of 2021 are taken because the industry classification results of listed companies for the fourth quarter of 2021 are not yet announced by the China Securities Regulatory Commission at the time of the deadline. Source: http://www.csrc.gov.cn/csrc/c100103/common_list.shtml.
5. Putting IMR into the second stage model may cause multicollinearity problems. Therefore, in the unreported results, we conducted VIF tests, and the results of VIFs are all less than 10, so there is no multicollinearity problem.
6. In the unlisted matching balance test, the results showed that the absolute value of the standardized deviation (% bias) of the matching variables was basically less than 5%, and the results of the inter-group t test were basically not significant, indicating that there was no systematic difference between the treatment group and the control group, the selection of covariates was appropriate, and the matching results were reliable.
7. The modified RI model is developed from the RI model of Li and Mohanram (2014) [66], and the RI model is modified by taking aggregate data instead of per-share data for regression model prediction to obtain the MRI model, which is consistent with the approach of Zou et al. (2019) [67].
8. The Bootstrap method is based on a repeated sampling approach. We conducted repeated sampling from the original sample to obtain a bootstrap sample similar to the original sample (Wen et al., 2010) [88]. Specifically, we obtained a bootstrap sample (with a capacity of 1000) by repeatedly sampling from the original sample in a put-back manner to obtain an estimate of the product of 1000 coefficients a and b. We then sorted these 1000 coefficient products from smallest to largest and then used those samples larger than 2.5% and smaller than 97.5% to form a confidence interval with 95% confidence. If the confidence interval does not contain 0, then the mediation effect is significant (Preacher et al., 2007) [89].
9. We performed the sample one-to-four matching in our previous robustness test and followed Abadie et al. (2004) [90], who found that a 1:4 matching of samples generally minimizes the mean squared error. Therefore, we conducted PSM 1:4 on sample pairings of different characteristics of share repurchases to treat the sample in order to minimize the external noise and endogeneity issues effects of the sample matching. We found no significant difference between the main sample matching variables of the experimental and control groups after the sample matching, hence meeting the parallelism hypothesis condition. These results were not reported in the paper due to its layout.
10. In addition to the apparently disclosed repurchase purpose, companies may also have potential repurchase purposes, inform the market about their share repurchase process, and change their purpose of repurchase during the process of implementation. These possibilities were not considered in this paper for the time being based on actual data.

## Supporting information

S1 Appendix (DOCX)
