# Supplementary material for: Share repurchase and the cost of capital: Discussion on the nature of share repurchase of Chinese listed companies
Source: PLoS One. 2023 Sep 28;18(9):e0292171. doi: 10.1371/journal.pone.0292171 (PMC10538758; doi:10.1371/journal.pone.0292171)
Supplement: S1 Appendix — (DOCX) [file pone.0292171.s001.docx]

Appendix

**Table A1. Main variable definition**

| **Variable type** | **symbol** | **Variable** | **Calculation and description** |
| --- | --- | --- | --- |
| Dependent variable | $R_{e}$ | The cost of capital | Take the average of six implied cost of capital estimation methods |
| Independent variable | *Rep_Dummy* | Share repurchase | Dummy variable: if the firm announces share repurchase programs, the value is 1, otherwise 0. |
|  | *Rep_Ratio* | Share repurchase ratio | The number of shares to be repurchased divided by the total number of shares outstanding. |
| Intermediary variable | *Analyst* | Information environment | The natural logarithm of the number of analysts followed plus 1 |
|  | *Amihud* | Information efficiency | The non–liquidity index, the formula is shown in the model (14) in the text |
| Control variables | *Size* | Firm size | Natural logarithm of total assets at the end of the period |
|  | *Mtb* | Market to book ratio | Total market value/book value of equity |
|  | *Beta* | Market risk | Beta value |
|  | *Growth* | Firm growth ability | Natural logarithm of the P/E ratio |
|  | *Lev* | firm leverage | The ratio of total debt to total assets. |
|  | *Oprisk* | Operating risk | Standard deviation of the change in ROA over the last three years |
|  | *Return* | stock return volatility | Annual individual stock returns considering reinvestment of cash dividends |
|  | *Cash* | Cash holdings | cash and cash equivalents/total assets |
|  | *Industry* | Industry fixed effect | Dummy variable: according to the 21 industries in the industry classification guidelines issued by the CSRC in the third quarter of 2021, the first two codes are used as the classification standard for manufacturing, and the first code is used as the classification standard for non-manufacturing industries |
|  | *Year* | Year fixed effect | Dummy variable: if the financial data belongs to a certain year from 2011 to 2021, the value of that year is 1; otherwise, it is 0 |

**Table A2. Analysis of the purpose of share repurchases**

| Year | **Cancellation of equity incentive** | | | | **Implement equity incentives** | | | |
| --- | --- | --- | --- | --- | --- | --- | --- | --- |
|  | **Freq.** | **Percent** | **Number** | **Amount** | **Freq.** | **Percent** | **Number** | **Amount** |
| 2011 | 0 | 0.000% | 0.000 | 0.000 | 0 | 0.000% | 0.000 | 0.000 |
| 2012 | 16 | 0.311% | 0.115 | 0.700 | 0 | 0.000% | 0.000 | 0.000 |
| 2013 | 91 | 1.769% | 1.026 | 4.598 | 6 | 0.117% | 1.639 | 6.570 |
| 2014 | 180 | 3.499% | 1.323 | 6.517 | 4 | 0.078% | 0.896 | 4.900 |
| 2015 | 241 | 4.685% | 2.217 | 11.410 | 5 | 0.097% | 0.383 | 6.182 |
| 2016 | 392 | 7.621% | 5.102 | 21.735 | 5 | 0.097% | 2.736 | 26.223 |
| 2017 | 540 | 10.498% | 5.335 | 35.727 | 30 | 0.583% | 4.764 | 55.320 |
| 2018 | 843 | 16.388% | 15.081 | 86.869 | 244 | 4.743% | 46.737 | 341.679 |
| 2019 | 957 | 18.604% | 11.443 | 80.496 | 180 | 3.499% | 43.687 | 408.322 |
| 2020 | 920 | 17.885% | 13.815 | 67.852 | 164 | 3.188% | 43.144 | 443.718 |
| 2021 | 964 | 18.740% | 10.765 | 66.089 | 285 | 5.540% | 50.439 | 765.099 |
| Total | 5144 | 72.176% | 66.222 | 381.993 | 923 | 12.951% | 194.425 | 2058.013 |
| Year | Market value management | | | | Others | | | |
|  | Freq. | Percent | Number | Amount | Freq. | Percent | Number | Amount |
| 2011 | 3 | 0.058% | 0.487 | 4.989 | 1 | 0.019% | 0.013 | 0.000 |
| 2012 | 14 | 0.272% | 13.612 | 66.784 | 1 | 0.019% | 0.042 | 0.000 |
| 2013 | 10 | 0.194% | 1.845 | 14.361 | 2 | 0.039% | 0.100 | 0.000 |
| 2014 | 6 | 0.117% | 0.579 | 4.139 | 13 | 0.253% | 24.775 | 68.578 |
| 2015 | 37 | 0.719% | 3.322 | 44.618 | 22 | 0.428% | 2.018 | 4.383 |
| 2016 | 7 | 0.136% | 7.169 | 34.222 | 44 | 0.855% | 2.426 | 0.000 |
| 2017 | 9 | 0.175% | 1.665 | 18.588 | 54 | 1.050% | 8.330 | 6.323 |
| 2018 | 107 | 2.080% | 21.634 | 192.041 | 144 | 2.799% | 26.204 | 120.177 |
| 2019 | 43 | 0.836% | 12.826 | 90.274 | 210 | 4.082% | 59.073 | 287.360 |
| 2020 | 34 | 0.661% | 31.284 | 178.241 | 125 | 2.430% | 22.031 | 63.428 |
| 2021 | 44 | 0.855% | 15.630 | 134.641 | 130 | 2.527% | 27.340 | 147.407 |
| Total | 314 | 4.406% | 110.053 | 782.898 | 746 | 10.467% | 172.352 | 697.656 |
